# Supplementary material for: Protocol for an ‘efficient design’ cluster randomised controlled trial to evaluate a complex intervention to improve antibiotic prescribing for CHIldren presenting to primary care with acute COugh and respiratory tract infection: the CHICO study
Source: BMJ Open. 2021 Mar 29;11(3):e041769. doi: 10.1136/bmjopen-2020-041769 (PMC8009213; doi:10.1136/bmjopen-2020-041769)
Supplement: Author's manuscript [file bmjopen-2020-041769.draft_revisions.pdf]

BMJ Open is committed to open peer review. As part of this commitment we make the peer review history of every article we publish publicly available.

When an article is published we post the peer reviewers' comments and the authors' responses online. We also post the versions of the paper that were used during peer review. These are the versions that the peer review comments apply to.

The versions of the paper that follow are the versions that were submitted during the peer review process. They are not the versions of record or the final published versions. They should not be cited or distributed as the published version of this manuscript.

BMJ Open is an open access journal and the full, final, typeset and author-corrected version of record of the manuscript is available on our site with no access controls, subscription charges or pay-per-view fees (http://bmjopen.bmj.com).

If you have any questions on BMJ Open's open peer review process please email info.bmjopen@bmj.com

BMJ Open

Protocol for an 'efficient design' cluster randomised controlled trial to evaluate a complex intervention to improve antibiotic prescribing for CHIldren presenting to primary care with acute COugh and respiratory tract infection: The CHICO study

Journal:	*BMJ Open*
Manuscript ID	bmjopen-2020-041769
Article Type:	Protocol
Date Submitted by the Author:	16-Jun-2020

Complete List of Authors:	Seume, Penny; University of Bristol, Centre for Academic Primary Care, Bristol Medical School: Population Health Sciences<br>Bevan, Scott; University of Bristol, Centre for Academic Primary Care, Bristol Medical School: Population Health Sciences<br>Young, Grace; University of Bristol, Bristol Trials Centre (Bristol Randomised Trial Collaboration), Bristol Medical School, University of Bristol<br>Ingram, Jenny; University of Bristol, Centre for Academic Child Health, Bristol Medical School, Population Health Sciences, University of Bristol<br>Clement, Clare; University of Bristol, Bristol Trials Centre (Bristol Randomised Trial Collaboration), Bristol Medical School, University of Bristol<br>Cabral, Christie; University of Bristol, Centre for Academic Primary Care, Bristol Medical School: Population Health Sciences, University of Bristol.<br>Lucas, Patricia; Unversity of Bristol, School for Policy Studies<br>Beech, Elizabeth; NHS Improvement<br>Taylor, Jodi; University of Bristol, Bristol Trials Centre (Bristol Randomised Trial Collaboration), Bristol Medical School, University of Bristo<br>Horwood, Jeremy; University of Bristol, UK, Centre for Academic Primary Care, Bristol Medical School: Population Health Sciences, University of Bristol.<br>Dixon, Padraig; University of Bristol, Centre for Academic Primary Care, Bristol Medical School: Population Health Sciences, University of Bristol.<br>Gulliford, Martin; King's College London, UK,<br>Francis, Nick; University of Southampton, School of Primary Care Population Sciences and Medical Education<br>Creavin, Sam T; University of Bristol, Centre for Academic Primary Care, Bristol Medical School: Population Health Sciences, University of Bristol.<br>Lane, Athene; University of Bristol, Bristol Trials Centre (Bristol Randomised Trial Collaboration), Bristol Medical School, University of Bristol<br>Hay, Alastair; University of Bristol, Centre for Academic Primary Care, Bristol Medical School: Population Health Sciences, University of Bristol.<br>Blair, Peter; University of Bristol, Centre for Academic Child Health, Bristol Medical School, Population Health Sciences, University of Bristol

Keywords:	Respiratory infections < THORACIC MEDICINE, Community child health < PAEDIATRICS, PRIMARY CARE, PUBLIC HEALTH

SCHOLARONE™ Manuscripts

I, the Submitting Author has the right to grant and does grant on behalf of all authors of the Work (as defined in the below author licence), an exclusive licence and/or a non-exclusive licence for contributions from authors who are: i) UK Crown employees; ii) where BMJ has agreed a CC-BY licence shall apply, and/or iii) in accordance with the terms applicable for US Federal Government officers or employees acting as part of their official duties; on a worldwide, perpetual, irrevocable, royalty-free basis to BMJ Publishing Group Ltd ("BMJ") its licensees and where the relevant Journal is co-owned by BMJ to the co-owners of the Journal, to publish the Work in this journal and any other BMJ products and to exploit all rights, as set out in our licence.

The Submitting Author accepts and understands that any supply made under these terms is made by BMJ to the Submitting Author unless you are acting as an employee on behalf of your employer or a postgraduate student of an affiliated institution which is paying any applicable article publishing charge ("APC") for Open Access articles. Where the Submitting Author wishes to make the Work available on an Open Access basis (and intends to pay the relevant APC), the terms of reuse of such Open Access shall be governed by a Creative Commons licence – details of these licences and which Creative Commons licence will apply to this Work are set out in our licence referred to above.

Other than as permitted in any relevant BMJ Author's Self Archiving Policies, I confirm this Work has not been accepted for publication elsewhere, is not being considered for publication elsewhere and does not duplicate material already published. I confirm all authors consent to publication of this Work and authorise the granting of this licence.

For peer review only

Protocol for an 'efficient design' cluster randomised controlled trial to evaluate a complex intervention to improve antibiotic prescribing for CHIldren presenting to primary care with acute COugh and respiratory tract infection: The CHICO study

Penny Seume[1], Scott Bevan[2], Grace Young[2], Jenny Ingram[3], Clare Clement[2], Christie Cabral[1], Patricia J Lucas[4], Elizabeth Beech[5], Jodi Taylor[2], Jeremy Horwood[1], Padraig Dixon[1], Martin Gulliford[6], Nick A Francis[7], Sam Creavin[1], Athene J. Lane[2], Alastair Hay[1] & Pete S. Blair[3]

Author Affiliations

[1] Centre for Academic Primary Care, Bristol Medical School: Population Health Sciences, University of Bristol.

[2] Bristol Trials Centre (Bristol Randomised Trial Collaboration), Bristol Medical School, University of Bristol

[3] Centre for Academic Child Health, Bristol Medical School, Population Health Sciences, University of Bristol

[4] School for Policy Studies, University of Bristol

[5] Patient Safety Team, NHS Improvement

[6] Kings College, University of London

[7] School of Primary Care Population Sciences and Medical Education, University of Southampton

Corresponding author:

Prof Peter S Blair (Professor of Epidemiology and Statistics), Centre for Academic Child Health, University of Bristol.

Address: Level D, St Michael's Hospital, Southwell St, Bristol, BS2 8EG

Phone: 01173425145 Fax: 01173425154 E-mail: p.s.blair@bris.ac.uk

Word Count: 3993/4000 words

Key words: Respiratory Tract Infection, Antibiotic Prescribing, Primary Care, Efficient Design

For peer review only

For peer review only - http://bmjopen.bmj.com/site/about/guidelines.xhtml

Article summary section

Abstract

Introduction

Respiratory tract infections (RTIs) in children are common and present major resource implications for primary care. Unnecessary use of antibiotics is associated with the development and proliferation of antimicrobial resistance. In 2016 the NIHR-funded 'TARGET' programme developed a prognostic algorithm to identify children with acute cough and RTI at very low risk of 30-day hospitalisation and unlikely to need antibiotics. The intervention includes: i) explicit elicitation of parental concerns, ii) the results of the prognostic algorithm accompanied by prescribing guidance and iii) provision of a printout for carers including safety netting advice. The CHICO (CHIldren's COugh) feasibility study suggested differential recruitment of healthier patients in control practices. This phase III 'efficiently designed' trial uses routinely collected data at the practice level, thus avoiding individual patient consent. The aim is to assess whether embedding a multi-faceted intervention into GP practice IT systems will result in reductions of antibiotic prescribing without impacting on hospital attendance for RTI.

Methods and Analysis

The co-primary outcomes are i) practice rate of dispensed amoxicillin and macrolide antibiotics ii) hospital admission rate for RTI using routinely collected data by Clinical Commissioning Groups (CCGs). Data will be collected for children aged 0-9 years registered at 310 practices (155 intervention, 155 usual care) over a 12-month period. Recruitment and randomisation of practices (using the EMISweb data management system) is conducted via each CCG stratified for children registered and baseline dispensing rates of each practice. Secondary outcomes will explore intervention effect modifiers. Qualitative interviews will explore intervention usage. The economic evaluation will be limited to a between-arm comparison in a cost-consequence analysis.

Ethics and Dissemination

Research ethics approval was given by London-Camden and Kings Cross Research Ethics Committee (ref:18/LO/0345). This manuscript refers to protocol version 4.0. Results will be disseminated through peer-reviewed journals and international conferences.

Trial Registration Number: ISRCTN11405239. This contains all items required to comply with the World Health Organization Trial Registration Data Set

For peer review only - http://bmjopen.bmj.com/site/about/guidelines.xhtml

Strengths and Limitations of this study

Strengths of this study

- Informed by a feasibility study this 'efficient-design' cluster RCT uses routinely collected aggregated measures for the co-primary outcomes, and avoids post-randomisation recruitment bias associated with individual patient consent
- The study will recruit practices across England thus including research-naïve practices and those serving diverse socio-economic populations
- The complex intervention, embedded within practice electronic health records, stems from a 5-year NIHR funded programme and includes: (i) a prognostic algorithm to stratify children's risk of hospitalisation due to respiratory infection in the following 30 days; (ii) tools to improve patient-doctor communication; and (iii) home care information (an alternate treatment action for clinicians)

Limitations of this study

- The design only allows for dispensing to be related to the number of children registered at the practice rather than the number consulting for in full RTI, and it will not allow quantification of delayed prescribing
- The other primary outcome is hospitalisation for RTI, and this relies on the quality of the data collected in full by CCGs - any difficulties obtaining this information or limitations of this efficient design will be reported

For peer review only

For peer review only

Introduction

Background

Acute respiratory tract infections (RTI) in children are a common reason for antibiotic prescribing. In English primary care, most antibiotics are prescribed for conditions that only sometimes require antibiotic treatment, depending on patient-specific indicators.[1] Although there has been a decline in prescribing for uncomplicated RTI in England over the last decade, more than a third of children were still prescribed antibiotics for these infections.[2] Clinical uncertainty in primary care regarding the prognosis of children with RTIs (i.e. knowing which children will and won't subsequently deteriorate) leads to the unnecessary use of existing antibiotics, which, combined with the slowing in development of new antibiotics, is associated with increasing antimicrobial resistance.[3,4] Qualitative work from our five year NIHR-funded 'TARGET' programme grant, completed in 2016, identified this uncertainty as a major driver of antibiotic prescribing.[5] We hypothesised that improved identification of children at very low risk of future hospitalisation might help reduce clinical uncertainty?[6] As part of the 'TARGET' programme we developed a prognostic algorithm that could be used by clinicians to identify children at very low risk of hospitalisation as well as tools to improve patient-doctor communication.[7]

Lessons learnt from the feasibility cluster RCT

Findings from across the 'TARGET' programme were used to develop a complex intervention designed to reduce antibiotic prescribing. The subsequent feasibility cluster randomised controlled trial [RCT] for CHIldren's Cough (CHICO) showed prescribing reductions in both arms of the trial but also exposed the differential recruitment of healthier children in the control arm.[8] In the qualitative interviews, clinicians reported preferential recruitment of less unwell children as these were quicker to manage and therefore easier to recruit. To negate differential recruitment, and conserve resources, an 'efficient design' was proposed for the full trial. Efficient design trials often utilise routinely collected data.[9] In the case of CHICO using aggregated data, this both avoids the need for individual patient consent (and differential recruitment) and utilises existing practice level data. The primary outcomes are routinely collected antibiotic dispensing data, collected by ePACT2 for the NHS prescribing services,[10] and hospital admission data collected by all English CCGs. Lessons learnt from the feasibility study also suggested better use of the tool would be facilitated if the intervention was embedded within the practice electronic health record system. The intervention in this study has thus been embedded in the EMIS (Egton Medical Information Systems) electronic patient record system, used in 56% of the primary care practices in England.[11]

For peer review only - http://bmjopen.bmj.com/site/about/guidelines.xhtml

Aims and Objectives

The aim of the CHICO RCT is to reduce antibiotic prescribing amongst children presenting with acute cough and RTI without increasing hospital admission for this condition.

The objectives are to determine whether the CHICO intervention decrease the number of dispensed prescriptions for oral amoxicillin and macrolide antibiotics for children aged 0-9 years presenting with acute cough and respiratory tract infections (efficacy comparison) and to determine if the CHICO intervention does not increase hospital admissions for children with a hospital diagnosis of RTI (non-inferiority comparison).

Methods and Analysis

Study Design

The CHICO RCT is an efficient, pragmatic open label, two-arm (intervention vs. usual care) trial with an embedded qualitative study, aimed at reducing antibiotic prescribing amongst children presenting with acute cough and RTI, with randomisation at the practice level, using routine antibiotic dispensing and hospitalisation data to assess effectiveness.

Study population, setting and recruitment plan

The study population is children aged 0-9 years presenting with acute cough and RTI. The setting is consultations in primary care practices with prescribing clinicians in diverse regions across England. Recruitment is at the practice level, so consent is not required for individual participants. Recruitment of practices is via CCGs using the Clinical Research Network (CRN) for support. All CCGs are already committed to national AMR strategies and an initial approach to several CCGs about collaboration in this study has been enthusiastically welcomed. CCGs with 15 or more EMIS practices will be targeted and we will use a member of the CCG medicines management team as the primary contact given the established links they already have helping to provide routine data.

Eligibility

Inclusion

GP practices in England using the EMIS electronic patient record system where the local CCG has agreed to provide data and the practice consented to take part.

Exclusion

Practices will be asked directly whether they are participating in any antimicrobial stewardship activities during our study period and these will be recorded. If these activities involve concurrent intervention studies where there is potential to confound or modify the effects of the intervention

For peer review only - http://bmjopen.bmj.com/site/about/guidelines.xhtml

these practices will be excluded. Practices involved in the CHICO feasibility study or are merging or planning to merge with another practice will also be excluded.

Treatment arms

Intervention

The theory-informed intervention[12] consists of both a clinician-focused algorithm to predict risk of hospitalisation for RTI in the following 30 days, in children with acute cough and RTI, and a carer-focused personalised printout recording decisions made at the consultation and safety netting information.[13]

The algorithm contains seven predictors (mnemonic STARWAVe): Short illness duration (parent/carer reported ≤3 days); raised Temperature (parent/carer reported severe in previous 24 hours or ≥37.8°C on examination); Age of child (<2 years); intercostal or subcostal Recession on examination; Wheeze during chest stethoscope examination; history of Asthma; and Vomiting (parent/carer reported moderate or severe in the 24 hours prior to consultation). The actions related to the algorithm scores are shown in Box 1, in each case the algorithm result (e.g. Low risk group) automatically appears and the pop-up text is available if the clinician hovers over the result. The algorithm is intended as a supportive additional component of a consultation in which it is likely that a number of aspects will inform the clinical decision making, including whether or not to prescribe antibiotics.

We will enrol a champion (e.g. a GP, nurse or practice manager) at each practice to help encourage and monitor the use of the intervention. These champions will help set up the intervention and run monthly queries of intervention use via EMIS that will be monitored centrally by the study team.

Training for practitioners in the intervention arm

The intervention clinicians will be provided with print and on-line evidence-based information to describe why, how and when to use the intervention. A practice champion will distribute the self-directed training materials within the practice and encourage all clinicians to use the intervention appropriately. In the training package for clinicians it will be emphasised that the primary purpose of the intervention is to support the care of the larger proportion of children (69%) who have a very low risk of hospitalisation.

Usual care

The clinicians in practices randomised to the comparator arm will be asked to treat children presenting with acute cough and RTI as they would normally. Baseline and follow-up data on control

For peer review only - http://bmjopen.bmj.com/site/about/guidelines.xhtml

practices will be collected but no data are being collected directly from the clinicians, no practice champions identified or specific contact being made.

Data collection and randomisation

Data collection takes place when both the individual practice and allied CCG agree to participate. Data will be entered onto a purpose designed database, validation and cleaning will be carried out throughout the trial. Only the administrative team and analysts will be able to access this data.

The number of dispensed amoxicillin and macrolides antibiotics given to children aged 0-9 years will be taken from the routine data source, epact2,[10] which provides practice-specific information by each 5-year age epoch. Data will be collected from CCGs for every participating practice with regards to the number of hospitalisations and emergency department attendances for respiratory tract infections. Only fully anonymised data sets will be sent from the GP practices and CCGs. This will be sent to a secure NHS e-mail address. We will collect data for the 12-month period each practice will be in the study and the 12-month period prior to randomisation. An 'implementation period' of around one month will allow time for the practices to install the intervention and encourage staff to use it. Any data collected during this period will not be used in the analysis. Where data is suppressed, owing to a low number of events, practices will be asked to provide aggregate 12-month data for baseline and follow up. Practice list size data, per month and 5-year epoch, will be obtained from the NHS digital website. In the unlikely event that a practice no longer wishes to participate, we will request all outstanding data collected up until the point of withdrawal. For intervention practices only, monthly intervention usage data will be captured. The data will be extracted from the EMIS system and will include how often the intervention is being used and by whom. Fidelity will be measured from the analysis of intervention data usage, scrutiny of the follow-up questionnaires and qualitative interviews.

The trial is supported by the Bristol Randomised Trials Collaboration (BRTC). The trial will conform to the BRTC standard operating procedures. The BRTC central research team will help prepare the trial documentation and data collection forms, specify the randomisation scheme, develop and maintain the study database, check data quality, monitor recruitment and carry out analyses in collaboration with the investigators. Both an independent Trials Steering Committee (TSC) and Data Monitoring Committee (DMC) will be appointed.

Baseline measurements

All GP practices recruited will be asked to complete a baseline questionnaire prior to randomisation to allow capture of practice characteristics. This includes: (i) practice staff composition (GP partners/salaried/sessional nurse practitioners and practice nurses and locums used in the last 12

For peer review only - http://bmjopen.bmj.com/site/about/guidelines.xhtml

months); (ii) available characteristics (such as postcode, total patients registered); (iii) registered child patients - number, age group, ethnicity and gender; (iv) triage systems used to handle children presenting with acute cough and respiratory tract infection; and (v) which clinicians prescribe antibiotics to children aged 0-9 years.

Randomisation

GP practices will be randomised on a 1:1 basis by the independent BRTC. Randomisation of practices will be stratified by CCG, with further minimisation by practice list size and baseline dispensing rates of 0-9 year olds; calculated using data from the 12 months prior to the CCG joining the CHICO study. A trial schematic is shown in Box 2.

Follow-up measurements

A follow-up questionnaire will be sent to all practices after 12 months (similar to the baseline questionnaire) asking about staffing levels and management of RTI amongst children as well as use of intervention for those in the intervention arm. Questions will also be included about whether the practice has merged or split with another practice, if they have had any related fatalities in children aged 0-9 years during the 12 months participation and for intervention practices only, their experience of using the intervention, problems encountered and whether they would use it again.

Blinding

As this is a cluster randomised controlled trial and due to the nature of the intervention delivery, it will not be possible to blind the practices to their allocation of either control or intervention group. Administrative staff will have access to individual data items, for entry into the database. The statistician will have access to aggregate information, by arm, to be able to report to the DMC and monitor hospitalisations.

Outcomes

The primary and secondary outcomes are listed in Box 3. All practices will collect data over a 12-month period, thus any seasonal fluctuations will be captured.

Safety reporting

Adverse events (AE) and serious adverse events (SAE) will be recorded and reported in accordance with Good Clinical Practice guidelines and the Sponsor's Research Related Adverse Event Reporting Policy. This trial is a low risk study, SAEs will only be reported if they are fatal or serious AND potentially related to trial participation (i.e. they result from advice provided by the intervention algorithm). As one of the outcomes for the trial is hospitalisation, we do expect some participants to be admitted to hospital (due to a deterioration of their underlying illness). Hospitalisation due to RTI

For peer review only - http://bmjopen.bmj.com/site/about/guidelines.xhtml

is an expected SAE and will not be subject to expedited reporting. Both SAEs and hospitalisation rates will be regularly reported to the DMC who will raise any safety concerns to the trial team and TSC for further action. Expected SAEs include but are not limited to pneumonia, empyema, deteriorating bronchiolitis.

SAEs related to the use of intervention

If the GP practice champion or attending clinician suspects that an SAE resulted from use of the intervention it should be reported to the study team immediately. The causality of the event will be assessed by the practice clinician and a delegated clinician working within the CHICO study team. If the event is deemed to be probably or definitely related to the intervention the SAE will be reported to the Research Ethics Committee (REC) and sponsor according to the expedited timescales.

Fatal SAEs

All practices should inform the study team immediately of any fatal SAEs in children that had presented with RTI at a practice consultation and were 0-9 years old at the time of consultation. This applies to any deaths occurring within 90 days of the consultation.

Internal pilot study

An internal pilot phase lasting 3 months and using 4 or more CCGs to recruit 60 practices will help establish how many CCGs we will eventually need to approach. Stop-go (traffic light) criteria will be used for i) practice recruitment, ii) identification of a practice champion, iii) intervention use and iv) ability to obtain dispensing data from the CCGs. A green light will be given for 80+% success (90% for dispensing data) and an amber light to implement remedial action at 70 to 79% (80-89% for dispensing data). A red light would indicate either a further pilot is needed or stopping the trial.

Sample size determination

Both sample size calculations assume 90% power and a conservative two-sided alpha of 0.025 to take account of the two co-primary outcomes. Both sample sizes also assume an intra-cluster correlation coefficient of 0.03 (which has been described as the upper confidence interval for ICCs in efficient cluster randomised trials[14,15]), an estimated coefficient of variation of 0.65 (to take account of differences in cluster size[16]) and an assumption of 750 children on average aged 0-9 years registered per practice (based on Bristol & Bath CCG data). Expected differences assumed: (i) a reduction in dispensing rate from 33 prescriptions per 100 registered children aged 0 to 9 years to 29 (or fewer) prescriptions (i.e. ≥10% overall reduction); and (ii) a hospitalisation rate that is no more than 2% in the intervention arm, compared with the control arm which is estimated to be 1%. This is based on a non-inferiority margin of 1%, however the investigators wanted to err on the side of

For peer review only - http://bmjopen.bmj.com/site/about/guidelines.xhtml

caution and use a two-sided alpha for the sample size calculation. This gave an overall sample size requirement of 310 practices; 155 intervention and 155 control practices.

Economic evaluation

To address our secondary aims (S2) a focus on costs will clarify whether and by how much NHS costs might change in the event of a widespread deployment of the algorithm into routine clinical practice Given the light-touch efficient design of the trial, the economic evaluation will be limited to a between-arm comparison of mean NHS costs in a cost-consequence analysis. NHS costs will be calculated from the costs of the intervention itself, prescriptions of amoxicillin and macrolides per the co-primary outcome, ED attendances and hospital admissions.

Qualitative study

Qualitative interviews with clinicians (GPs and practice nurses) and other practice staff (managers, pharmacists) and CCG staff (medicines managers) will explore the use of the intervention, how it was embedded into practice and whether it was used appropriately. The interview topic guide will be informed by Normalisation Process Theory (NPT) developed to explain the social processes leading to routine embedding of complex interventions in health care.[17,18]

NPT proposes that implementation of interventions is dependent on the ability of participants to fulfil four criteria; 'coherence' (how people make sense of the intervention), 'cognitive participation' (the work to develop new practices), 'collective action' (the work to operationalise practices), and 'reflexive monitoring' (ways in which people appraise how new practices are working).

Clinicians and other key staff from the intervention practices will be invited to participate in semi-structured interviews to explore their views and experiences of the intervention. Audio recorded verbal consent will be taken from participants. The first set of interviews will be conducted during the internal pilot phase and findings fed back to help guide best practice during the rest of the study. A second phase of interviews will be conducted when the clinicians have been using the intervention for several months to investigate the normalisation and sustainability of using the intervention. Interviews are expected to take 30-45 minutes.

Purposive sampling will be used to include a maximum variation sample to take account of: clinical experience, dispensing rates of practices and practices serving areas of high and low social-economic deprivation. The sample sizes will be determined by the need to achieve data saturation, such that no new themes are emerging from the data by the end of data collection.[19] Interviews will be analysed in batches. This is likely to include up to 30 clinicians and 20 other staff involved in implementation.

For peer review only - http://bmjopen.bmj.com/site/about/guidelines.xhtml

Data analysis

Quantitative data analysis

All analyses and reporting will be in line with CONSORT guidelines and its extension for cluster randomised trials.[20] Primary analyses will be conducted on an intention-to-treat (ITT) basis, a per protocol analysis will also be conducted as part of the sensitivity analyses . A full CHICO statistical analysis plan will be developed and agreed by the TSC prior to undertaking analyses of the main trial. The statistical analysis plan will include health economics and qualitative analysis subsections. At the end of the trial, all outcomes will be described and compared with the appropriate descriptive statistics where relevant: mean and standard deviation (SD) for continuous and count outcomes, medians and inter-quartile range if required for skewed data and numbers and percentages for dichotomous and categorical outcomes. Depending on the dispersion of the data we may use linear regression or a random effects Poisson regression (negative binomial regression) model to analyse both co-primaries, with CCG included as a random effect. This has the advantage of incorporating person/years follow up (number of children at a practice multiplied by the length of follow-up for that practice) and examining clustering by CCG. Each co-primary will be adjusted for baseline dispensing rates or hospitalisation rates, using the 12 months of data collected prior to randomisation. Effects of number of practices within CCGs and number of patients within each practice will also be investigated in a sensitivity analysis. Other baseline characteristics between practices will be examined to ensure randomisation is balanced in the two arms. Any differences in excess of 0.5 SDs or 10% or more will be controlled for in sensitivity analyses to ensure that the imbalance does not affect the overall result. The effects of missing data will be explored using sensitivity analyses. We anticipate no more than 10% missing data and that it will be missing at random. The pattern and extent of missing data will be explored and any changes to the methods described in the analysis plan will be fully justified in the study report and publication. All quantitative data will be analysed using STATA.

Qualitative data analysis

Interviews will be transcribed and anonymised. Analysis will inform further data collection: for instance, analytic insights from data gathered in earlier interviews will help identify any changes that need to be made to the topic guides during later interviews. Qualitative analysis of the transcripts will follow recognised thematic analysis procedures using NVivo software.[21] Thematic analysis,[22] utilising a data-driven inductive approach,[23] will be used to scrutinise the data in order to identify and analyse patterns and themes of particular salience for participants and across the dataset.[24]

For peer review only - http://bmjopen.bmj.com/site/about/guidelines.xhtml

Patient and public involvement

This intervention has been developed collaboratively with our parent advisory group (PAG) and clinical advisory group (CAG) throughout the 'TARGET' programme. Their comments and suggestions about the format of the intervention and parent/carer materials have informed both the intervention and the design of the earlier feasibility study.

Similar involvement will be sought for the trial. We will seek agreement from a newly formed PAG to meet throughout the study to report on progress of the study and discuss issues that arise during the study. PAG members will input into all the materials for parents/carers as they are further developed including any patient-facing tools. We will also form a clinician and pharmacist advisory group (CPAG) to assist with the implementation and any further refinements to the intervention. They will meet once in person and then contribute by Skype or email to refine GP information and intervention delivery.

Study duration and timeline

The initial duration was 33 months from 1st March 2018 to 30th November 2020 although a subsequent extension of 12 months has been awarded to extend the study to 30th November 2021 to recruit the target number of practices. The timeline includes study set up (8 months), internal pilot (3 months), recruitment of practices via CCGs (15 months), follow-up of data collection (12 months) and analysis (7 months).

Study Management

The study will be monitored and audited in accordance with the Sponsor's policy, which is consistent with the UK Policy Framework for Health and Social Care Research. The following data monitor checks will be carried out by the co-ordination team; that data collected are consistent with adherence to the study protocol; that CRFs are only being completed by authorised persons; that SAE recording and reporting procedures are being followed correctly; that no key data are missing and that data are valid.

Trial oversight

The study is overseen by a Trial Management Group that meet on a monthly basis and consist of the Chief Investigator (CI), grant holders, study sponsor and any other staff responsible for the delivery of the trial. The TSC provide independent supervision of the trial and oversees trial progress. The TSC consists of an independent chair (GP and Clinical Academic) and four other independent members including a statistician, a second Clinician and two PPI representative, as well as the CI. The DMC

For peer review only - http://bmjopen.bmj.com/site/about/guidelines.xhtml

monitors patient safety and trial data efficacy and consists of an independent chair, two other independent members, the CI and trial statistician.

All SAE's are recorded and notified as appropriate to the relevant authorities. The University of Bristol is acting as sponsor for this trial and is responsible for overall oversight of the trial.

Ethics and Dissemination

Ethics

We are not recruiting individual patients to this study and the primary outcome data are already collected routinely thus we do not need patient consent. We will consent the individual practices and encourage all clinicians in the intervention practices to use the intervention tool appropriately. The intervention is directed at the clinician primarily to change their prescribing behaviour. Any data collected from individual clinicians will be anonymised. The personalised letter given to the patients will not contain information on risk of hospitalisation, but rather details of the consultation and the usual safe-guarding information. The CHICO RCT falls under the remit of draft guidance[25] for 'simple and efficient trials' due to the nature of the intervention and the low level of risk involved for patients and meets the suggested principles provided by NHS Health Research Authority (HRA).[26]

Dissemination

A comprehensive plan for disseminating CHICO results will be developed and outputs from this research will comply with the CHICO RCT publication policy and internationally accepted guidelines (CONSORT). The results of the study will be published in the academic press and all GP practices will be offered a lay summary of the main findings of the study. We will disseminate the findings both at a primary care level via CCGs and national conferences as well as international conferences. Whether or not the trial provides evidence of effect we will provide evidence of the potential benefits or pitfalls of an efficiently designed trial; including the utility of routine data collection; the capacity to collect data through current practice systems and the effectiveness of using practice champions and progress feedback to encourage use of such interventions.

For peer review only - http://bmjopen.bmj.com/site/about/guidelines.xhtml

Trial Status

Currently 261 EMIS GP practices have been greenlighted across 15 CRN regions in the UK. The first GP practice was recruited to the study in September 2018 , with recruitment currently ongoing.

Acknowledgements

This study was designed and delivered in collaboration with the Bristol Randomised Trials Collaboration (BRTC), part of the Bristol Trials Centre, is in receipt of National Institute for Health Research CTU support funding. The University of Bristol is acting as the sponsor for this trial and the trial is hosted by the NHS Bristol, North Somerset and South Gloucestershire Clinical Commissioning Group (CCG). The authors would like to thank all General practices, CCGs and CRNs for their involvement in CHICO. The authors would also like to thank the members of the TSC and DMC.

Author Contributions

AH, PB, PL, NF and JI were responsible for developing the research questions. PB, AH, JI, PL, CCab, CClem, EB, MG, JH, SC, AL and NF and are responsible for the study design and collection of data. PS and SB are responsible for study management and coordination. GY, PD and CClem are responsible for the analysis of the data. PB drafted the paper. All authors read, commented on and approved the final manuscript.

Public and patient involvement

This intervention has been developed collaboratively with our parent advisory group (PAG) and clinical advisory group (CAG) throughout the TARGET programme. Their comments and suggestions about the format of the intervention and parent/carer materials have informed both the intervention and the design of the earlier feasibility study. Similar involvement will be sought for the trial. We will seek agreement from a newly formed PAG to meet throughout the study, allowing the investigators to report on progress of the study and discuss issues that arise during the study. PAG members will input into all the materials for parents/carers as they are further developed including any patient-facing tools. We will also form a clinician and pharmacist advisory group (CPAG) to assist with the implementation and any further refining the intervention. They will meet once in person and then contribute by Skype or email to refine GP information and intervention delivery.

Funding

This research is funded by the National Institute for Health Research (NIHR) Health Technology Assessment (HTA) programme (funder ref: 16/31/98). The views expressed are those of the authors and not necessarily those of the NIHR or the Department of Health and Social Care.

For peer review only - http://bmjopen.bmj.com/site/about/guidelines.xhtml

Availability of data and materials

The datasets analysed during the current study will be available from the corresponding author on reasonable request.

Competing interest

None declared

Patient consent for publication

Not required.

Licence Statement

I, the Submitting Author has the right to grant and does grant on behalf of all authors of the Work (as defined in the below author licence), an exclusive licence and/or a non-exclusive licence for contributions from authors who are: i) UK Crown employees; ii) where BMJ has agreed a CC-BY licence shall apply, and/or iii) in accordance with the terms applicable for US Federal Government officers or employees acting as part of their official duties; on a worldwide, perpetual, irrevocable, royalty-free basis to BMJ Publishing Group Ltd ("BMJ") its licensees and where the relevant Journal is co-owned by BMJ to the co-owners of the Journal, to publish the Work in BMJ Open and any other BMJ products and to exploit all rights, as set out in our licence.

Ethics approval

The study received North of Scotland Research Ethics Committee (REC) approval on October 29th 2018 and Health Research Authority approval on November 14th 2018. Any amendments to the protocol will be reported accordingly to the regulatory bodies.

Author e-mail and ORCID IDs http://orcid.org/

Penny Seume (PS):	penny.seume@bristol.ac.uk	0000-0001-9407-5828
Scott Bevan (SB):	scott.t.bevan@gmail.com	0000-0002-4567-5111
Grace Young (GY):	grace.young@bristol.ac.uk	0000-0002-5210-1183
Jenny Ingram (JI):	jenny.ingram@bristol.ac.uk	0000-0003-2366-008X
Clare Clement (CClem):	c.clement@bristol.ac.uk	0000-0002-5555-433X
Christie Cabral (CCab):	christie.cabral@bristol.ac.uk	
Patricia Lucas (PL):	patricia.lucas@bristol.ac.uk	0000-0002-0469-8085
Elizabeth Beech (EB):	Elizabeth.beech@nhs.net	
Jodi Taylor (JT):	j.taylor@bristol.ac.uk	0000-0001-7171-8923

For peer review only - http://bmjopen.bmj.com/site/about/guidelines.xhtml

For peer review only

Jeremy Horwood (JH):	jj.horwood@bristol.ac.uk	0000-0001-7092-4960
Padraig Dixon (PD):	Padraig.dixon@bristol.ac.uk	0000-0001-5285-409X
Martin Gulliford (MG):	martin.gulliford@kcl.ac.uk	0000-0003-1898-9075
Nick Francis (NF):	nick.francis@soton.ac.uk	
Sam Creavin (SC) :	sam.creavin@bristol.ac.uk	0000-0002-6772-7111
Alastair Hay (AH):	alastair.hay@bristol.ac.uk	0000-0003-3012-375X
Athene Lane (AL):	athene.lane@bristol.ac.uk	0000-0002-7578-4925
Peter Blair (PB):	p.s.blair@bris.ac.uk	0000-0002-7832-8087

For peer review only

For peer review only - http://bmjopen.bmj.com/site/about/guidelines.xhtml

References

1. Smith DRM, Dolk FCK, Pouwels KB, Christie M, Robotham JV, Smieszek T. Defining the appropriateness and inappropriateness of antibiotic prescribing in primary care. J Antimicrob Chemother. 2018 Feb 1;73(suppl_2):ii11-ii18.
2. Bou-Antoun S, Costelloe C, Honeyford K, Mazidi M, Hayhoe BWJ, Holmes A, Johnson AP, Aylin P. Age-related decline in antibiotic prescribing for uncomplicated respiratory tract infections in primary care in England following the introduction of a national financial incentive (the Quality Premium) for health commissioners to reduce use of antibiotics in the community: an interrupted time series analysis. J Antimicrob Chemother. 2018 Oct 1;73(10):2883-2892.
3. Vodicka TA, Thompson M, Lucas P, Heneghan C, Blair PS, Buckley DI, Redmond N and Hay AD. Reducing antibiotic prescribing for children with respiratory tract infections in primary care: a systematic review. Br J Gen Pract 2013 Jul;63(612): e445-54.
4. Davies SC , Gibbens N . UK five year antimicrobial resistance strategy 2013 to 2018. London: Department of Health, Crown Copyright 2013. https://assets.publishing.service.gov.uk/government/uploads/system/uploads/attachment_data/file/244058/20130902_UK_5_year_AMR_strategy.pdf
5. Horwood, J, Cabral, C, Hay, A & Ingram, J, 2015, 'Primary healthcare practitioners' diagnostic and antibiotic prescribing decisions in children's consultations for respiratory tract infections'. British Journal of General Practice. 66 (644), e207-e213
6. Cabral C, Ingram J, Hay AD, Horwood J; TARGET team. "They just say everything's a virus"—Parent's judgment of the credibility of clinician communication in primary care consultations for respiratory tract infections in children: A qualitative study. Patient Educ Couns. 2014 May;95(2):248-53.
7. Hay AD, Redmond NM, Turnbull S, Christensen H, Thornton H, Little P, Thompson M, Delaney B, Lovering AM, Muir P, et al. Development and internal validation of a clinical rule to improve antibiotic use in children presenting to primary care with acute respiratory tract infection and cough: a prognostic cohort study. Lancet Respir Med. 2016;11:902-10.
8. Blair PS, Turnbull S, Ingram J, Redmond N, Lucas PJ, Cabral C, Hollinghurst S, Dixon P, Peters T, Horwood J, Little P, Francis NA, Gilbertson A, Jameson C, Hay AD. Feasibility cluster randomised controlled trial of a within-consultation intervention to reduce antibiotic prescribing for children presenting to primary care with acute respiratory tract infection and cough. BMJ Open. 2017 May 9;7(5):e014506.
9. NIHR: Annual Efficient Studies funding calls for CTU projects. https://www.nihr.ac.uk/documents/ad-hoc-funding-calls-for-ctu-projects/20141

For peer review only - http://bmjopen.bmj.com/site/about/guidelines.xhtml

10. ePACT2 prescribing data held by NHS Prescription Services. https://www.nhsbsa.nhs.uk/epact2
11. Kontopantelis E, Stevens RJ, Helms PJ, Edwards D, Doran T, Ashcroft DM. Spatial Distribution of Clinical Computer Systems in Primary Care in England in 2016 and Implications for Primary Care Electronic Medical Record Databases: A Cross-Sectional Population Study. BMJ Open. 2018 Feb 28;8(2):e020738.
12. Lucas PJ, Ingram J, Redmond NM, Cabral C, Turnbull SL, Hay AD. Development of an Intervention to Reduce Antibiotic Use for Childhood Coughs in UK Primary Care Using Critical Synthesis of Multi-Method Research. BMC Med Res Methodol 2017 Dec 28;17(1):175.
13. Cabral C, Ingram J, Redmond N, Horwood J, Blair P, Hollinghurst S, Hay A, Lucas P. Caring for children with coughs: Information and advice for parents. https://child-cough.bristol.ac.uk, University of Bristol, 2016.
14. Hemming K, Eldridge S, Forbes G et al. 2017. How to design efficient cluster randomised trials. BMJ 2017;358:j3064
15. Gulliford, M.C., van Staa, T.P., McDermott L. et al. 2014. Cluster randomized trials utilizing primary care electronic health records: methodological issues in design, conduct, and analysis (eCRT study).
16. Eldridge SM, Ashby D and Kerry S. Sample size for cluster randomized trials: effect of coefficient of variation of cluster size and analysis method. Int J of Epidemiology 2006;35:1292–1300
17. May CR, Mair F, Finch T, et al. Development of a theory of implementation and integration: Normalization Process Theory. Implementation Science: IS 2009; 4:29-29.
18. Murray E, Treweek S, Pope C, et al. Normalisation process theory: a framework for developing, evaluating and implementing complex interventions. BMC Medicine 2010; 8:63-63.
19. Sandelowski M. Sample size in qualitative research. Research in Nursing & Health 1995; 18(2):179-83.
20. Schulz KF, Altman DG, Moher D; CONSORT Group. CONSORT 2010 Statement: updated guidelines for reporting parallel group randomised trials. BMJ 2010;340:c332
21. QSR International. What is NVivo? | NVivo 2019 [Available from: https://www.qsrinternational.com/nvivo-qualitative-data-analysis-software/about/nvivo.
22. Braun V, Clarke V. Using thematic analysis in psychology. Qualitative Research in Psychology 2006; 3(2):77-101.

For peer review only - http://bmjopen.bmj.com/site/about/guidelines.xhtml

23. Boyatzis R. Transforming qualitative information: Thematic analysis and code development. Thousand Oaks, CA: Sage, 1998.
24. Charmay K. Constructing grounded theory: a practical guide through qualitative analysis. London: Sage, 2006.
25. Collett C. Seeking informed consent for simple and efficient trials in the NHS. Draft guidance and responses: Health Research Authority, February 2016.
26. Zwarenstein M, Treweek S, Gagnier J.J et al. Improving the reporting of pragmatic trials: an extension of the CONSORT statement. BMJ 2008, 337:a2390

For peer review only

Box 1: Text for algorithm result

Algorithm result	Pop-up text
Very low risk group	Very reassuring CHICO score: 0 or 1 CHICO predictors: >99.6% of children will recover from this illness with home care. Consider a no or delayed antibiotic prescribing strategy. CHICO leaflet and letter covers common concerns and safety netting advice.
Average risk group	Reassuring CHICO score: 2 or 3 CHICO predictors: >98% of children will recover from this illness with home care. Consider no or delayed antibiotic prescribing strategy. CHICO leaflet and letter covers common concerns and safety netting advice.
Elevated risk group	Safety netting needed: 4+ CHICO predictors: This is more than average, but >87% of children will still recover from this illness with home care. Highlight SAFETY NETTING advice in CHICO leaflet.

For peer review only

For peer review only - http://bmjopen.bmj.com/site/about/guidelines.xhtml

Box 2: Trial schematic

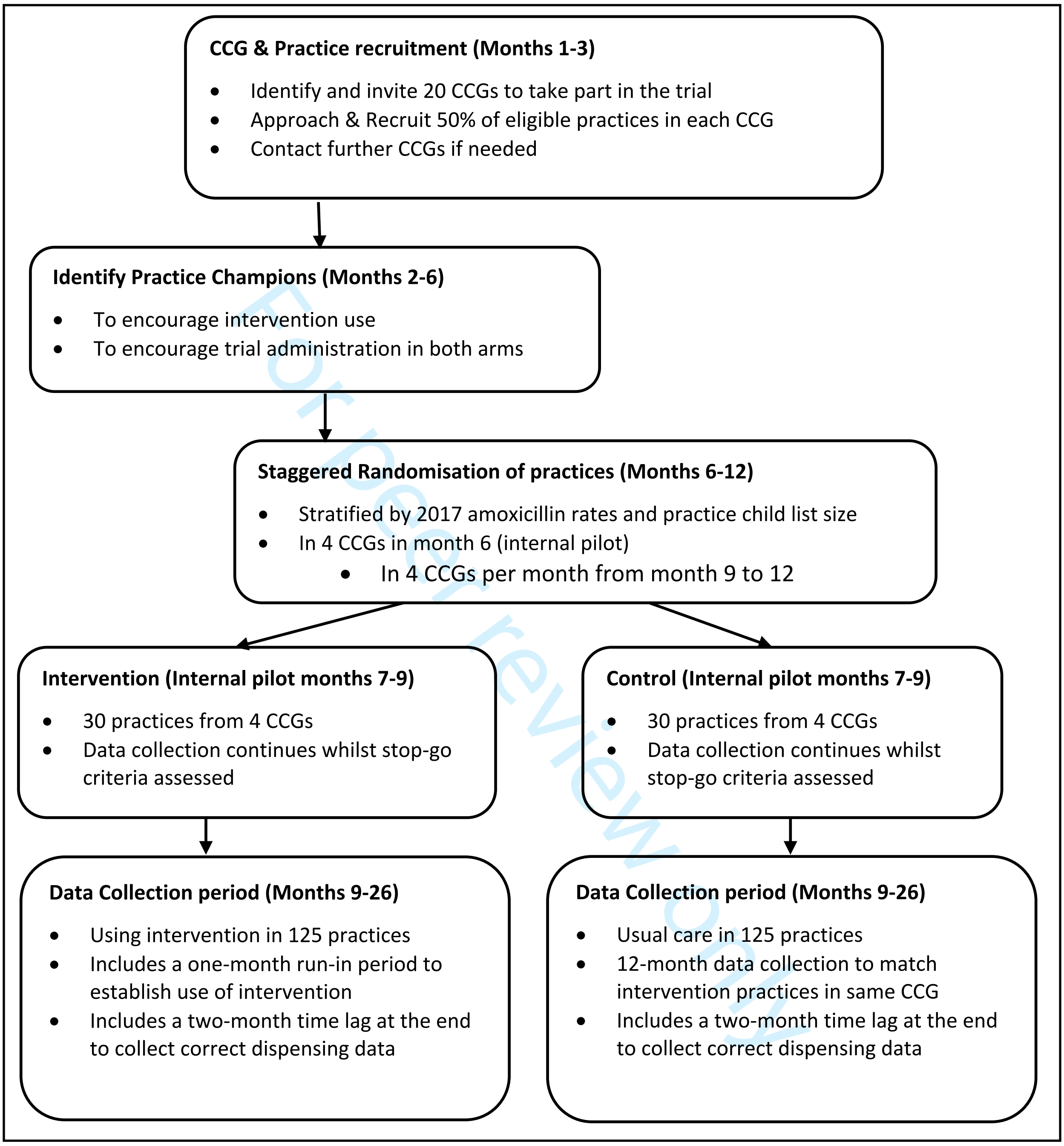

For peer review only

For peer review only - http://bmjopen.bmj.com/site/about/guidelines.xhtml

Box 3: Detailed Study Outcomes

**Primary Outcomes**	
P1)	Whether the CHICO intervention decrease the number of dispensed prescriptions for oral amoxicillin and macrolide antibiotics[1] for respiratory tract infections to children presenting with acute cough and respiratory tract infection to primary care (efficacy comparison).
P2)	Whether the CHICO intervention result in no increase in hospital admissions[2] for children with a hospital diagnosis of RTI (non-inferiority comparison).
**Secondary Outcomes**	
S1)	Whether the CHICO intervention results in no change in the Emergency Department (ED) attendance rates[3] of children with a diagnosis of RTI.
S2)	The costs to the NHS of using the CHICO intervention (health economic outcome).
S3)	Whether there is any intervention effect modified by the number of locums used in the practice (treatment interaction).
S4)	Whether there is any intervention effect modified by the practices' prior antibiotic prescribing rate (treatment interaction).
S5)	Whether the effects of the CHICO intervention differ between practices with or without nurse prescribers (treatment interaction)[4].
S6)	Whether the effects of the CHICO intervention differ between practices with 1 site versus multiple sites (branches) at each practice (treatment interaction).
S7)	Whether the effects of the CHICO intervention differ within child age groups.
S8)	Whether the use of the CHICO intervention varies between practices (adherence) and over time (seasonal differences) and the influence this has on the dispensing rates.
S9)	Whether the embedded CHICO intervention is acceptable to, and used by, primary care clinicians (GPs and practice nurses).

1 The dispensing rate, calculated by adding the number of amoxicillin and macrolide antibiotics dispensed over the follow up year divided by the number of children aged 0-9 years (median monthly list size) at each practice over the 12-month follow up period.
2. The rate of hospital admission for RTI amongst children aged 0-9 years using the same denominator as above.
3. This is a secondary outcome already collected from practices by CCGs.
4. If a large majority of practices have nurse prescribers then we may look at this as a continuous percentage of nurse prescribers, out of all GP and nurse prescribers.

For peer review only

SPIRIT
STANDARD PROTOCOL ITEMS: RECOMMENDATIONS FOR INTERVENTIONAL TRIALS

SPIRIT 2013 Checklist: Recommended items to address in a clinical trial protocol and related documents*

Section/item	Item No	Description	Addressed on page number
**Administrative information**			
Title	1	Descriptive title identifying the study design, population, interventions, and, if applicable, trial acronym	Title Page (p1)
Trial registration	2a	Trial identifier and registry name. If not yet registered, name of intended registry	4
	2b	All items from the World Health Organization Trial Registration Data Set	4
Protocol version	3	Date and version identifier	3
Funding	4	Sources and types of financial, material, and other support	15
Roles and responsibilities	5a	Names, affiliations, and roles of protocol contributors	1
	5b	Name and contact information for the trial sponsor	15
	5c	Role of study sponsor and funders, if any, in study design; collection, management, analysis, and interpretation of data; writing of the report; and the decision to submit the report for publication, including whether they will have ultimate authority over any of these activities	Not applicable
	5d	Composition, roles, and responsibilities of the coordinating centre, steering committee, endpoint adjudication committee, data management team, and other individuals or groups overseeing the trial, if applicable (see Item 21a for data monitoring committee)	8,10,12,13,14

For peer review only

**Introduction**			
Background and rationale	6a	Description of research question and justification for undertaking the trial, including summary of relevant studies (published and unpublished) examining benefits and harms for each intervention	Abstract (p3),5
	6b	Explanation for choice of comparators	7-8
Objectives	7	Specific objectives or hypotheses	6
Trial design	8	Description of trial design including type of trial (eg, parallel group, crossover, factorial, single group), allocation ratio, and framework (eg, superiority, equivalence, noninferiority, exploratory)	6
**Methods: Participants, interventions, and outcomes**			
Study setting	9	Description of study settings (eg, community clinic, academic hospital) and list of countries where data will be collected. Reference to where list of study sites can be obtained	6
Eligibility criteria	10	Inclusion and exclusion criteria for participants. If applicable, eligibility criteria for study centres and individuals who will perform the interventions (eg, surgeons, psychotherapists)	6-7
Interventions	11a	Interventions for each group with sufficient detail to allow replication, including how and when they will be administered	7
	11b	Criteria for discontinuing or modifying allocated interventions for a given trial participant (eg, drug dose change in response to harms, participant request, or improving/worsening disease)	Not Applicable
	11c	Strategies to improve adherence to intervention protocols, and any procedures for monitoring adherence (eg, drug tablet return, laboratory tests)	Not Applicable
	11d	Relevant concomitant care and interventions that are permitted or prohibited during the trial	6
Outcomes	12	Primary, secondary, and other outcomes, including the specific measurement variable (eg, systolic blood pressure), analysis metric (eg, change from baseline, final value, time to event), method of aggregation (eg, median, proportion), and time point for each outcome. Explanation of the clinical relevance of chosen efficacy and harm outcomes is strongly recommended	3,9,12,Box 3 (p22)
Participant timeline	13	Time schedule of enrolment, interventions (including any run-ins and washouts), assessments, and visits for participants. A schematic diagram is highly recommended (see Figure)	Box 2 (p21)

For peer review only - http://bmjopen.bmj.com/site/about/guidelines.xhtml

Sample size	14	Estimated number of participants needed to achieve study objectives and how it was determined, including clinical and statistical assumptions supporting any sample size calculations	10-11
Recruitment	15	Strategies for achieving adequate participant enrolment to reach target sample size	6,10
**Methods: Assignment of interventions (for controlled trials)**			
Allocation:			
Sequence generation	16a	Method of generating the allocation sequence (eg, computer-generated random numbers), and list of any factors for stratification. To reduce predictability of a random sequence, details of any planned restriction (eg, blocking) should be provided in a separate document that is unavailable to those who enrol participants or assign interventions	3,8-9
Allocation concealment mechanism	16b	Mechanism of implementing the allocation sequence (eg, central telephone; sequentially numbered, opaque, sealed envelopes), describing any steps to conceal the sequence until interventions are assigned	8,12
Implementation	16c	Who will generate the allocation sequence, who will enrol participants, and who will assign participants to interventions	8-9
Blinding (masking)	17a	Who will be blinded after assignment to interventions (eg, trial participants, care providers, outcome assessors, data analysts), and how	9
	17b	If blinded, circumstances under which unblinding is permissible, and procedure for revealing a participant's allocated intervention during the trial	Not applicable
**Methods: Data collection, management, and analysis**			
Data collection methods	18a	Plans for assessment and collection of outcome, baseline, and other trial data, including any related processes to promote data quality (eg, duplicate measurements, training of assessors) and a description of study instruments (eg, questionnaires, laboratory tests) along with their reliability and validity, if known. Reference to where data collection forms can be found, if not in the protocol	7-9
	18b	Plans to promote participant retention and complete follow-up, including list of any outcome data to be collected for participants who discontinue or deviate from intervention protocols	

Data management	19	Plans for data entry, coding, security, and storage, including any related processes to promote data quality (eg, double data entry; range checks for data values). Reference to where details of data management procedures can be found, if not in the protocol	13
Statistical methods	20a	Statistical methods for analysing primary and secondary outcomes. Reference to where other details of the statistical analysis plan can be found, if not in the protocol	12
	20b	Methods for any additional analyses (eg, subgroup and adjusted analyses)	12
	20c	Definition of analysis population relating to protocol non-adherence (eg, as randomised analysis), and any statistical methods to handle missing data (eg, multiple imputation)	12
**Methods: Monitoring**			
Data monitoring	21a	Composition of data monitoring committee (DMC); summary of its role and reporting structure; statement of whether it is independent from the sponsor and competing interests; and reference to where further details about its charter can be found, if not in the protocol. Alternatively, an explanation of why a DMC is not needed	10,13-14
	21b	Description of any interim analyses and stopping guidelines, including who will have access to these interim results and make the final decision to terminate the trial	Not applicable
Harms	22	Plans for collecting, assessing, reporting, and managing solicited and spontaneously reported adverse events and other unintended effects of trial interventions or trial conduct	9-10
Auditing	23	Frequency and procedures for auditing trial conduct, if any, and whether the process will be independent from investigators and the sponsor	10
**Ethics and dissemination**			
Research ethics approval	24	Plans for seeking research ethics committee/institutional review board (REC/IRB) approval	3
Protocol amendments	25	Plans for communicating important protocol modifications (eg, changes to eligibility criteria, outcomes, analyses) to relevant parties (eg, investigators, REC/IRBs, trial participants, trial registries, journals, regulators)	15

For peer review only - http://bmjopen.bmj.com/site/about/guidelines.xhtml

Consent or assent	26a	Who will obtain informed consent or assent from potential trial participants or authorised surrogates, and how (see Item 32)	Not applicable
	26b	Additional consent provisions for collection and use of participant data and biological specimens in ancillary studies, if applicable	Not applicable
Confidentiality	27	How personal information about potential and enrolled participants will be collected, shared, and maintained in order to protect confidentiality before, during, and after the trial	8
Declaration of interests	28	Financial and other competing interests for principal investigators for the overall trial and each study site	Not applicable
Access to data	29	Statement of who will have access to the final trial dataset, and disclosure of contractual agreements that limit such access for investigators	15
Ancillary and post-trial care	30	Provisions, if any, for ancillary and post-trial care, and for compensation to those who suffer harm from trial participation	Not applicable
Dissemination policy	31a	Plans for investigators and sponsor to communicate trial results to participants, healthcare professionals, the public, and other relevant groups (eg, via publication, reporting in results databases, or other data sharing arrangements), including any publication restrictions	14
	31b	Authorship eligibility guidelines and any intended use of professional writers	Not applicable
	31c	Plans, if any, for granting public access to the full protocol, participant-level dataset, and statistical code	Not applicable
**Appendices**			
Informed consent materials	32	Model consent form and other related documentation given to participants and authorised surrogates	Not applicable
Biological specimens	33	Plans for collection, laboratory evaluation, and storage of biological specimens for genetic or molecular analysis in the current trial and for future use in ancillary studies, if applicable	Not applicable

*It is strongly recommended that this checklist be read in conjunction with the SPIRIT 2013 Explanation & Elaboration for important clarification on the items. Amendments to the protocol should be tracked and dated. The SPIRIT checklist is copyrighted by the SPIRIT Group under the Creative Commons "Attribution-NonCommercial-NoDerivs 3.0 Unported" license.

For peer review only - http://bmjopen.bmj.com/site/about/guidelines.xhtml

BMJ Open

Protocol for an 'efficient design' cluster randomised controlled trial to evaluate a complex intervention to improve antibiotic prescribing for CHIldren presenting to primary care with acute COugh and respiratory tract infection: The CHICO study

Journal:	*BMJ Open*
Manuscript ID	bmjopen-2020-041769.R1
Article Type:	Protocol
Date Submitted by the Author:	18-Dec-2020

Complete List of Authors:	Seume, Penny; University of Bristol, Centre for Academic Primary Care, Bristol Medical School: Population Health Sciences<br>Bevan, Scott; University of Bristol, Centre for Academic Primary Care, Bristol Medical School: Population Health Sciences<br>Young, Grace; University of Bristol, Bristol Trials Centre (Bristol Randomised Trial Collaboration), Bristol Medical School, University of Bristol<br>Ingram, Jenny; University of Bristol, Centre for Academic Child Health, Bristol Medical School, Population Health Sciences, University of Bristol<br>Clement, Clare; University of Bristol, Bristol Trials Centre (Bristol Randomised Trial Collaboration), Bristol Medical School, University of Bristol<br>Cabral, Christie; University of Bristol, Centre for Academic Primary Care, Bristol Medical School: Population Health Sciences, University of Bristol.<br>Lucas, Patricia; Unversity of Bristol, School for Policy Studies<br>Beech, Elizabeth; NHS Improvement<br>Taylor, Jodi; University of Bristol, Bristol Trials Centre (Bristol Randomised Trial Collaboration), Bristol Medical School, University of Bristo<br>Horwood, Jeremy; University of Bristol, UK, Centre for Academic Primary Care, Bristol Medical School: Population Health Sciences, University of Bristol.<br>Dixon, Padraig; University of Bristol, Centre for Academic Primary Care, Bristol Medical School: Population Health Sciences, University of Bristol.<br>Gulliford, Martin; King's College London, UK,<br>Francis, Nick; University of Southampton, School of Primary Care Population Sciences and Medical Education<br>Creavin, Sam T; University of Bristol, Centre for Academic Primary Care, Bristol Medical School: Population Health Sciences, University of Bristol.<br>Lane, Athene; University of Bristol, Bristol Trials Centre (Bristol Randomised Trial Collaboration), Bristol Medical School, University of Bristol<br>Hay, Alastair; University of Bristol, Centre for Academic Primary Care, Bristol Medical School: Population Health Sciences, University of Bristol.<br>Blair, Peter; University of Bristol, Centre for Academic Child Health, Bristol Medical School, Population Health Sciences, University of Bristol

**Primary Subject Heading**:	General practice / Family practice
Secondary Subject Heading:	Respiratory medicine, Evidence based practice
Keywords:	Respiratory infections < THORACIC MEDICINE, Community child health < PAEDIATRICS, PRIMARY CARE, PUBLIC HEALTH

SCHOLARONE™
Manuscripts

I, the Submitting Author has the right to grant and does grant on behalf of all authors of the Work (as defined in the below author licence), an exclusive licence and/or a non-exclusive licence for contributions from authors who are: i) UK Crown employees; ii) where BMJ has agreed a CC-BY licence shall apply, and/or iii) in accordance with the terms applicable for US Federal Government officers or employees acting as part of their official duties; on a worldwide, perpetual, irrevocable, royalty-free basis to BMJ Publishing Group Ltd ("BMJ") its licensees and where the relevant Journal is co-owned by BMJ to the co-owners of the Journal, to publish the Work in this journal and any other BMJ products and to exploit all rights, as set out in our licence.

The Submitting Author accepts and understands that any supply made under these terms is made by BMJ to the Submitting Author unless you are acting as an employee on behalf of your employer or a postgraduate student of an affiliated institution which is paying any applicable article publishing charge ("APC") for Open Access articles. Where the Submitting Author wishes to make the Work available on an Open Access basis (and intends to pay the relevant APC), the terms of reuse of such Open Access shall be governed by a Creative Commons licence – details of these licences and which Creative Commons licence will apply to this Work are set out in our licence referred to above.

Other than as permitted in any relevant BMJ Author's Self Archiving Policies, I confirm this Work has not been accepted for publication elsewhere, is not being considered for publication elsewhere and does not duplicate material already published. I confirm all authors consent to publication of this Work and authorise the granting of this licence.

For peer review only

Protocol for an 'efficient design' cluster randomised controlled trial to evaluate a complex intervention to improve antibiotic prescribing for CHIldren presenting to primary care with acute COugh and respiratory tract infection: The CHICO study

Penny Seume[1], Scott Bevan[2], Grace Young[2], Jenny Ingram[3], Clare Clement[2], Christie Cabral[1], Patricia J Lucas[4], Elizabeth Beech[5], Jodi Taylor[2], Jeremy Horwood[1], Padraig Dixon[6], Martin Gulliford[7], Nick A Francis[8], Sam Creavin[1], Athene J. Lane[2], Alastair D Hay[1] & Pete S. Blair[3]

For peer review only

Author Affiliations

[1] Centre for Academic Primary Care, Bristol Medical School: Population Health Sciences, University of Bristol.

[2] Bristol Trials Centre (Bristol Randomised Trial Collaboration), Bristol Medical School, University of Bristol

[3] Centre for Academic Child Health, Bristol Medical School, Population Health Sciences, University of Bristol

[4] School for Policy Studies, University of Bristol

[5] NHS England and NHS Improvement, South West Region

[6] Nuffield Department of Primary Care Health Sciences, University of Oxford. Email: padraig.dixon@phc.ox.ac.uk

[7] Kings College, University of London

[8] School of Primary Care Population Sciences and Medical Education, University of Southampton

Corresponding author:

Prof Peter S Blair (Professor of Epidemiology and Statistics), Centre for Academic Child Health, University of Bristol.

Address: Level D, St Michael's Hospital, Southwell St, Bristol, BS2 8EG

Phone: 01173425145 Fax: 01173425154 E-mail: p.s.blair@bris.ac.uk

Word Count: 4083/4000 words

Key words: Respiratory Tract Infection, Antibiotic Prescribing, Primary Care, Efficient Design

For peer review only

For peer review only - http://bmjopen.bmj.com/site/about/guidelines.xhtml

Article summary section

Abstract

Introduction

Respiratory tract infections (RTIs) in children are common and present major resource implications for primary care. Unnecessary use of antibiotics is associated with the development and proliferation of antimicrobial resistance. In 2016 the NIHR-funded 'TARGET' programme developed a prognostic algorithm to identify children with acute cough and RTI at very low risk of 30-day hospitalisation and unlikely to need antibiotics. The intervention includes: i) explicit elicitation of parental concerns, ii) the results of the prognostic algorithm accompanied by prescribing guidance and iii) provision of a printout for carers including safety netting advice. The CHICO (CHIldren's COugh) feasibility study suggested differential recruitment of healthier patients in control practices. This phase III 'efficiently designed' trial uses routinely collected data at the practice level, thus avoiding individual patient consent. The aim is to assess whether embedding a multi-faceted intervention into GP practice IT systems will result in reductions of antibiotic prescribing without impacting on hospital attendance for RTI.

Methods and Analysis

The co-primary outcomes are i) practice rate of dispensed amoxicillin and macrolide antibiotics ii) hospital admission rate for RTI using routinely collected data by Clinical Commissioning Groups (CCGs). Data will be collected for children aged 0-9 years registered at 310 practices (155 intervention, 155 usual care) over a 12-month period. Recruitment and randomisation of practices (using the EMISweb data management system) is conducted via each CCG stratified for children registered and baseline dispensing rates of each practice. Secondary outcomes will explore intervention effect modifiers. Qualitative interviews will explore intervention usage. The economic evaluation will be limited to a between-arm comparison in a cost-consequence analysis.

Ethics and Dissemination

Research ethics approval was given by London-Camden and Kings Cross Research Ethics Committee (ref:18/LO/0345). This manuscript refers to protocol version 4.0. Results will be disseminated through peer-reviewed journals and international conferences.

Trial Registration Number: ISRCTN11405239. This contains all items required to comply with the World Health Organization Trial Registration Data Set

For peer review only

Strengths and Limitations of this study

Strengths of this study

- Informed by a feasibility study this 'efficient-design' cluster RCT uses routinely collected aggregated measures for the co-primary outcomes, and avoids post-randomisation recruitment bias associated with individual patient consent
- The study will recruit practices across England thus including research-naïve practices and those serving diverse socio-economic populations
- The complex intervention, embedded within practice electronic health records, stems from a 5-year NIHR funded programme and includes: (i) a prognostic algorithm to stratify children's risk of hospitalisation due to respiratory infection in the following 30 days; (ii) tools to improve patient-doctor communication; and (iii) home care information (an alternate treatment action for clinicians)

Limitations of this study

- The design only allows for dispensing to be related to the number of children registered at the practice rather than the number consulting for in full RTI, and it will not allow quantification of delayed prescribing
- The other primary outcome is hospitalisation for RTI, and this relies on the quality of the data collected in full by CCGs - any difficulties obtaining this information or limitations of this efficient design will be reported

For peer review only

For peer review only - http://bmjopen.bmj.com/site/about/guidelines.xhtml

Introduction

Background

Acute respiratory tract infections (RTI) in children are a common reason for antibiotic prescribing. In English primary care, most antibiotics are prescribed for conditions that only sometimes require antibiotic treatment, depending on patient-specific indicators.[1] Although there has been a decline in prescribing for uncomplicated RTI in England over the last decade, more than a third of children were still prescribed antibiotics for these infections.[2] Clinical uncertainty in primary care regarding the prognosis of children with RTIs (i.e. knowing which children will and won't subsequently deteriorate) contributes to the unnecessary use of existing antibiotics, which is associated with increasing antimicrobial resistance.[3,4] Qualitative work from our five year NIHR-funded 'TARGET' programme grant, completed in 2016, identified this uncertainty as a major driver of antibiotic prescribing.[5] We hypothesised that improved identification of children at very low risk of future hospitalisation might help reduce clinical uncertainty.[6] As part of the 'TARGET' programme we developed a prognostic algorithm that could be used by clinicians to identify children at very low risk of hospitalisation as well as tools to improve patient-doctor communication.[7]

Lessons learnt from the feasibility cluster RCT

Findings from across the 'TARGET' programme were used to develop a complex intervention designed to reduce antibiotic prescribing. The subsequent feasibility cluster randomised controlled trial [RCT] for CHIldren's COugh (CHICO) showed significant prescribing reductions in both arms of the trial compared to the cohort data of the programme but also exposed both lower prescribing levels and differential recruitment of healthier children in the control arm.[8] In the qualitative interviews, clinicians reported preferential recruitment of less unwell children as these were quicker to manage and therefore easier to recruit. To negate differential recruitment, and conserve resources, an 'efficient design' was proposed for the full trial. Efficient design trials often utilise routinely collected data.[9] In the case of CHICO using aggregated data, this both avoids the need for individual patient consent (and differential recruitment) and utilises existing practice level data. This simpler design, placing fewer demands on clinicians and practices compared to other studies, will also encourage the recruitment of research-naïve practices. The primary outcomes are routinely collected antibiotic dispensing data, collected by ePACT2 for the NHS prescribing services,[10] and hospital admission data collected by all English Clinical Commissioning Groups (CCGs decide what services are needed for diverse local populations, and ensure that they are provided (https://www.england.nhs.uk/ccgs/). They also hold responsibility for local antimicrobial prescribing guidelines.. Lessons learnt from the feasibility study also suggested better use of the tool would be

For peer review only - http://bmjopen.bmj.com/site/about/guidelines.xhtml

facilitated if the intervention was embedded within the practice electronic health record system. The intervention in this study has thus been embedded in the EMIS (*Egton Medical Information Systems*) electronic patient record system, used in 56% of the primary care practices in England.[11]

Aims and Objectives

The aim of the CHICO RCT is to reduce antibiotic prescribing amongst children presenting with acute cough and RTI without increasing hospital admission for this condition.

The objectives are to determine whether the CHICO intervention decrease the number of dispensed prescriptions for oral amoxicillin and macrolide antibiotics (the predominant antibiotics given to children presenting with acute cough and respiratory tract infections in the UK) for children aged 0-9 years (efficacy comparison) and to determine if the CHICO intervention does not increase hospital admissions for children with a hospital diagnosis of RTI (non-inferiority comparison).

Methods and Analysis

Study Design

The CHICO RCT is an efficient, pragmatic open label, two-arm (intervention vs. usual care) trial with an embedded qualitative study, aimed at reducing antibiotic prescribing amongst children presenting with acute cough and RTI, with randomisation at the practice level, using routine antibiotic dispensing and hospitalisation data to assess effectiveness.

Study population, setting and recruitment plan

The study population is children aged 0-9 years presenting with acute cough and RTI. Oral suspensions are more often given to this age group. The setting is consultations in primary care practices with prescribing clinicians in diverse regions across England. Recruitment is at the practice level, so consent is not required for individual participants. Recruitment of practices is via CCGs and by using the Clinical Research Network (CRN) who support patients, the public and health and care organisations across England to participate in high-quality research (https://www.nihr.ac.uk/explore-nihr/support/clinical-research-network.htm). All CCGs are already committed to national AMR strategies and an initial approach to several CCGs about collaboration in this study has been enthusiastically welcomed. CCGs with 15 or more EMIS practices will be targeted and we will use a member of the CCG medicines management team as the primary contact given the established links they already have helping to provide routine data.

For peer review only - http://bmjopen.bmj.com/site/about/guidelines.xhtml

For peer review only

Eligibility

Inclusion

GP practices in England using the EMIS electronic patient record system where the local CCG has agreed to provide data and the practice consented to take part.

Exclusion

Practices will be asked directly whether they are participating in any antimicrobial stewardship activities during our study period and these will be recorded. If these activities involve concurrent intervention studies where there is potential to confound or modify the effects of the intervention these practices will be excluded. Practices involved in the CHICO feasibility study or are merging or planning to merge with another practice will also be excluded.

Treatment arms

Intervention

The theory-informed intervention[12] consists of both a clinician-focused algorithm to predict risk of hospitalisation for RTI in the following 30 days, in children with acute cough and RTI, and carer-focused personalised information recording decisions made at the consultation and safety netting information.[13]

The algorithm contains seven predictors (mnemonic STARWAVe): Short illness duration (parent/carer reported ≤3 days); raised Temperature (parent/carer reported severe in previous 24 hours or ≥37.8°C on examination); Age of child (<2 years); intercostal or subcostal Recession on examination; Wheeze during chest stethoscope examination; history of Asthma; and Vomiting (parent/carer reported moderate or severe in the 24 hours prior to consultation). The actions related to the algorithm scores are shown in Table 1, in each case the algorithm result (e.g. Low risk group) automatically appears and the pop-up text is available if the clinician hovers over the result. The algorithm is intended as a supportive additional component of a consultation in which it is likely that a number of aspects will inform the clinical decision making, including whether or not to prescribe antibiotics.

We will enrol a champion (e.g. a GP, nurse or practice manager) at each practice to help encourage and monitor the use of the intervention. These champions will help set up the intervention and run monthly queries of intervention use via EMIS that will be monitored centrally by the study team.

Training for practitioners in the intervention arm

The intervention clinicians will be provided with print and on-line evidence-based information to describe why, how and when to use the intervention. A practice champion will distribute the self-

For peer review only - http://bmjopen.bmj.com/site/about/guidelines.xhtml

directed training materials within the practice and encourage all clinicians to use the intervention appropriately. In the training package for clinicians it will be emphasised that the primary purpose of the intervention is to support the care of the larger proportion of children (69%) who have a very low risk of hospitalisation.

Usual care

The clinicians in practices randomised to the comparator arm will be asked to treat children presenting with acute cough and RTI as they would normally. Baseline and follow-up data on control practices will be collected but no data are being collected directly from the clinicians, no practice champions identified or specific contact being made.

Patient and public involvement

This intervention has been developed collaboratively with our parent advisory group (PAG) and clinical advisory group (CAG) throughout the 'TARGET' programme. Their comments and suggestions about the format of the intervention and parent/carer materials have informed both the intervention and the design of the earlier feasibility study.

Similar involvement will be sought for the trial. We will seek agreement from a newly formed PAG to meet throughout the study to report on progress of the study and discuss issues that arise during the study. PAG members will input into all the materials for parents/carers as they are further developed including any patient-facing tools. We will also form a clinician and pharmacist advisory group (CPAG) to assist with the implementation and any further refinements to the intervention. They will meet once in person and then contribute by Skype or email to refine GP information and intervention delivery.

Data collection and randomisation

Data collection takes place when both the individual practice and allied CCG agree to participate. Data will be entered onto a purpose designed database, validation and cleaning will be carried out throughout the trial. Only the administrative team and analysts will be able to access this data.

The number of dispensed amoxicillin and macrolides antibiotics given to children aged 0-9 years will be taken from the routine data source, epact2,[10] which provides practice-specific information by each 5-year age epoch. Data will be collected from CCGs for every participating practice with regards to the number of hospitalisations and emergency department attendances for respiratory tract infections. Only fully anonymised data sets will be sent from the GP practices and CCGs. This will be sent to a secure NHS e-mail address. We will collect data for the 12-month period each practice will be in the study and the 12-month period prior to randomisation. An 'implementation period' of

For peer review only - http://bmjopen.bmj.com/site/about/guidelines.xhtml

around one month will allow time for the practices to install the intervention and encourage staff to use it. Any data collected during this period will not be used in the analysis. Where data is suppressed, owing to a low number of events, practices will be asked to provide aggregate 12-month data for baseline and follow up. Practice list size data, per month and 5-year epoch, will be obtained from the NHS digital website. In the unlikely event that a practice no longer wishes to participate, we will request all outstanding data collected up until the point of withdrawal. For intervention practices only, monthly intervention usage data will be captured. The data will be extracted from the EMIS system and will include how often the intervention is being used and by whom. Fidelity will be measured from the analysis of intervention data usage, scrutiny of the follow-up questionnaires and qualitative interviews.

The trial is supported by the Bristol Randomised Trials Collaboration (BRTC). The trial will conform to the BRTC standard operating procedures. The BRTC central research team will help prepare the trial documentation and data collection forms, specify the randomisation scheme, develop and maintain the study database, check data quality, monitor recruitment and carry out analyses in collaboration with the investigators. Both an independent Trials Steering Committee (TSC) and Data Monitoring Committee (DMC) will be appointed.

Baseline measurements

All GP practices recruited will be asked to complete a baseline questionnaire prior to randomisation to allow capture of practice characteristics. This includes: (i) practice staff composition (GP partners/salaried/sessional nurse practitioners and practice nurses and locums used in the last 12 months); (ii) available characteristics (such as postcode, total patients registered); (iii) registered child patients - number, age group, ethnicity and gender; (iv) triage systems used to handle children presenting with acute cough and respiratory tract infection; and (v) which clinicians prescribe antibiotics to children aged 0-9 years.

Randomisation

GP practices will be randomised on a 1:1 basis by the independent BRTC. Randomisation of practices will be stratified by CCG, with further minimisation by practice list size and baseline dispensing rates of 0-9 year olds; calculated using data from the 12 months prior to the CCG joining the CHICO study. A trial schematic is shown in Figure 1.

Follow-up measurements

A follow-up questionnaire will be sent to all practices after 12 months (similar to the baseline questionnaire) asking about staffing levels and management of RTI amongst children as well as use of intervention for those in the intervention arm. Questions will also be included about whether the

For peer review only - http://bmjopen.bmj.com/site/about/guidelines.xhtml

For peer review only

practice has merged or split with another practice, if they have had any related fatalities in children aged 0-9 years during the 12 months participation and for intervention practices only, their experience of using the intervention, problems encountered and whether they would use it again.

Blinding

As this is a cluster randomised controlled trial and due to the nature of the intervention delivery, it will not be possible to blind the practices to their allocation of either control or intervention group. Administrative staff will have access to individual data items, for entry into the database. The statistician will have access to aggregate information, by arm, to be able to report to the DMC and monitor hospitalisations.

Outcomes

The primary and secondary outcomes are listed in Table 2. All practices will collect data over a 12-month period, thus any seasonal fluctuations will be captured.

Safety reporting

Adverse events (AE) and serious adverse events (SAE) will be recorded and reported in accordance with Good Clinical Practice guidelines and the Sponsor's Research Related Adverse Event Reporting Policy. This trial is a low risk study, SAEs will only be reported if they are fatal or serious AND potentially related to trial participation (i.e. they result from advice provided by the intervention algorithm). As one of the outcomes for the trial is hospitalisation, we do expect some participants to be admitted to hospital (due to a deterioration of their underlying illness). Hospitalisation due to RTI is an expected SAE and will not be subject to expedited reporting. Both SAEs and hospitalisation rates will be regularly reported to the DMC who will raise any safety concerns to the trial team and TSC for further action. Expected SAEs include but are not limited to pneumonia, empyema, deteriorating bronchiolitis.

SAEs related to the use of intervention

If the GP practice champion or attending clinician suspects that an SAE resulted from use of the intervention it should be reported to the study team immediately. The causality of the event will be assessed by the practice clinician and a delegated clinician working within the CHICO study team. If the event is deemed to be probably or definitely related to the intervention the SAE will be reported to the Research Ethics Committee (REC) and sponsor according to the expedited timescales.

For peer review only

Fatal SAEs

All practices should inform the study team immediately of any fatal SAEs in children that had presented with RTI at a practice consultation and were 0-9 years old at the time of consultation. This applies to any deaths occurring within 90 days of the consultation.

Internal pilot study

An internal pilot phase lasting 3 months and using 4 or more CCGs to recruit 60 practices will help establish how many CCGs we will eventually need to approach. Stop-go (traffic light) criteria will be used for i) practice recruitment, ii) identification of a practice champion, iii) intervention use and iv) ability to obtain dispensing data from the CCGs. A green light will be given for 80+% success (90% for dispensing data) and an amber light to implement remedial action at 70 to 79% (80-89% for dispensing data). A red light would indicate either a further pilot is needed or stopping the trial.

Sample size determination

Both sample size calculations assume 90% power and a conservative two-sided alpha of 0.025 to take account of the two co-primary outcomes. Both sample sizes also assume an intra-cluster correlation coefficient of 0.03 (which has been described as the upper confidence interval for ICCs in efficient cluster randomised trials[14,15]), an estimated coefficient of variation of 0.65 (to take account of differences in cluster size[16]) and an assumption of 750 children on average aged 0-9 years registered per practice (based on Bristol & Bath CCG data). Expected differences assumed: (i) a reduction in dispensing rate from 33 prescriptions per 100 registered children aged 0 to 9 years to 29 (or fewer) prescriptions (i.e. ≥10% overall reduction); and (ii) a hospitalisation rate that is no more than 2% in the intervention arm, compared with the control arm which is estimated to be 1%. This is based on a non-inferiority margin of 1%, however the investigators wanted to err on the side of caution and use a two-sided alpha for the sample size calculation. This gave an overall sample size requirement of 310 practices; 155 intervention and 155 control practices.

Economic evaluation

To address our secondary aims (S2) a focus on costs will clarify whether and by how much NHS costs might change in the event of a widespread deployment of the algorithm into routine clinical practice. Given the light-touch efficient design of the trial, the economic evaluation will be limited to a between-arm comparison of mean NHS costs in a cost-consequence analysis. NHS costs will be calculated from the costs of the intervention itself, prescriptions of amoxicillin and macrolides per the co-primary outcome, ED attendances and hospital admissions.

For peer review only - http://bmjopen.bmj.com/site/about/guidelines.xhtml

Qualitative study

Qualitative interviews with clinicians (GPs and practice nurses) and other practice staff (managers, pharmacists) and CCG staff (medicines managers) will explore the use of the intervention, how it was embedded into practice and whether it was used appropriately. The interview topic guide will be informed by Normalisation Process Theory (NPT) developed to explain the social processes leading to routine embedding of complex interventions in health care.[17,18]

NPT proposes that implementation of interventions is dependent on the ability of participants to fulfil four criteria; 'coherence' (how people make sense of the intervention), 'cognitive participation' (the work to develop new practices), 'collective action' (the work to operationalise practices), and 'reflexive monitoring' (ways in which people appraise how new practices are working).

Clinicians and other key staff from the intervention practices will be invited to participate in semi-structured interviews to explore their views and experiences of the intervention. Audio recorded verbal consent will be taken from participants. The first set of interviews will be conducted during the internal pilot phase and findings fed back to help guide best practice during the rest of the study. A second phase of interviews will be conducted when the clinicians have been using the intervention for several months to investigate the normalisation and sustainability of using the intervention. Interviews are expected to take 30-45 minutes.

Purposive sampling will be used to include a maximum variation sample to take account of: clinical experience, dispensing rates of practices and practices serving areas of high and low social-economic deprivation. The sample sizes will be determined by the need to achieve data saturation, such that no new themes are emerging from the data by the end of data collection.[19] Interviews will be analysed in batches. This is likely to include up to 30 clinicians and 20 other staff involved in implementation.

Data analysis

Quantitative data analysis

All analyses and reporting will be in line with CONSORT guidelines and its extension for cluster randomised trials.[20] Primary analyses will be conducted on an intention-to-treat (ITT) basis, a per protocol analysis will also be conducted as part of the sensitivity analyses . A full CHICO statistical analysis plan will be developed and agreed by the TSC prior to undertaking analyses of the main trial. The statistical analysis plan will include health economics and qualitative analysis subsections. At the end of the trial, all outcomes will be described and compared with the appropriate descriptive statistics where relevant: mean and standard deviation (SD) for continuous and count outcomes, medians and inter-quartile range if required for skewed data and numbers and percentages for

For peer review only - http://bmjopen.bmj.com/site/about/guidelines.xhtml

For peer review only

dichotomous and categorical outcomes. Depending on the dispersion of the data we may use linear regression or a random effects Poisson regression (negative binomial regression) model to analyse both co-primaries, with CCG included as a random effect. This has the advantage of incorporating person-years follow up (number of children at a practice multiplied by the length of follow-up for that practice) and examining clustering by CCG. Each co-primary will be adjusted for baseline dispensing rates or hospitalisation rates, using the 12 months of data collected prior to randomisation. Effects of number of practices within CCGs and number of patients within each practice will also be investigated in a sensitivity analysis. Other baseline characteristics between practices will be examined to ensure randomisation is balanced in the two arms. Any differences in excess of 0.5 SDs or 10% or more will be controlled for in sensitivity analyses to ensure that the imbalance does not affect the overall result. The effects of missing data will be explored using sensitivity analyses. We anticipate no more than 10% missing data and that it will be missing at random. The pattern and extent of missing data will be explored and any changes to the methods described in the analysis plan will be fully justified in the study report and publication. All quantitative data will be analysed using Stata.

Qualitative data analysis

Interviews will be transcribed and anonymised. Analysis will inform further data collection: for instance, analytic insights from data gathered in earlier interviews will help identify any changes that need to be made to the topic guides during later interviews. Qualitative analysis of the transcripts will follow recognised thematic analysis procedures using NVivo software.[21] Thematic analysis,[22] utilising a data-driven inductive approach,[23] will be used to scrutinise the data in order to identify and analyse patterns and themes of particular salience for participants and across the dataset.[24]

Study duration and timeline

The initial duration was 33 months from 1st March 2018 to 30th November 2020 although a subsequent extension of 12 months has been awarded to extend the study to 30th November 2021 to recruit the target number of practices. The timeline includes study set up (8 months), internal pilot (3 months), recruitment of practices via CCGs (15 months), follow-up of data collection (12 months) and analysis (7 months).

Study Management

The study will be monitored and audited in accordance with the Sponsor's policy, which is consistent with the UK Policy Framework for Health and Social Care Research. The following data monitor checks will be carried out by the co-ordination team; that data collected are consistent with adherence to the study protocol; that CRFs are only being completed by authorised persons; that

For peer review only - http://bmjopen.bmj.com/site/about/guidelines.xhtml

SAE recording and reporting procedures are being followed correctly; that no key data are missing and that data are valid.

Trial oversight

The study is overseen by a Trial Management Group that meet on a monthly basis and consist of the Chief Investigator (CI), grant holders, study sponsor and any other staff responsible for the delivery of the trial. The TSC provide independent supervision of the trial and oversees trial progress. The TSC consists of an independent chair (GP and Clinical Academic) and four other independent members including a statistician, a second Clinician and two PPI representative, as well as the CI. The DMC monitors patient safety and trial data efficacy and consists of an independent chair, two other independent members, the CI and trial statistician.

All SAE's are recorded and notified as appropriate to the relevant authorities. The University of Bristol is acting as sponsor for this trial and is responsible for overall oversight of the trial.

Ethics and Dissemination

Ethics

We are not recruiting individual patients to this study and the primary outcome data are already collected routinely thus we do not need patient consent. We will consent the individual practices and encourage all clinicians in the intervention practices to use the intervention tool appropriately. The intervention is directed at the clinician primarily to change their prescribing behaviour. Any data collected from individual clinicians will be anonymised. The personalised letter given to the patients will not contain information on risk of hospitalisation, but rather details of the consultation and the usual safe-guarding information. The CHICO RCT falls under the remit of draft guidance[25] for 'simple and efficient trials' due to the nature of the intervention and the low level of risk involved for patients and meets the suggested principles provided by NHS Health Research Authority (HRA).[26]

Dissemination

A comprehensive plan for disseminating CHICO results will be developed and outputs from this research will comply with the CHICO RCT publication policy and internationally accepted guidelines (CONSORT). The results of the study will be published in the academic press and all GP practices will be offered a lay summary of the main findings of the study. We will disseminate the findings both at a primary care level via CCGs and national conferences as well as international conferences. Whether or not the trial provides evidence of effect we will provide evidence of the potential

For peer review only - http://bmjopen.bmj.com/site/about/guidelines.xhtml

benefits or pitfalls of an efficiently designed trial; including the utility of routine data collection; the capacity to collect data through current practice systems and the effectiveness of using practice champions and progress feedback to encourage use of such interventions.

Trial Status

Currently (July 2020) 261 EMIS GP practices have been greenlighted across 15 CRN regions in the UK. The first GP practice was recruited to the study in September 2018, with recruitment currently ongoing.

Acknowledgements

This study was designed and delivered in collaboration with the Bristol Randomised Trials Collaboration (BRTC), part of the Bristol Trials Centre, is in receipt of National Institute for Health Research CTU support funding. The University of Bristol is acting as the sponsor for this trial and the trial is hosted by the NHS Bristol, North Somerset and South Gloucestershire Clinical Commissioning Group (CCG). The authors would like to thank all General practices, CCGs and CRNs for their involvement in CHICO. The authors would also like to thank the members of the TSC and DMC.

Author Contributions

AH, PB, PL, NF and JI were responsible for developing the research questions. PB, AH, JI, PL, CCab, CClem, EB, MG, JH, SC, AL and NF and are responsible for the study design and collection of data. PS, JT and SB are responsible for study management and coordination. GY, PD and CClem are responsible for the analysis of the data. PB drafted the paper. All authors read, commented on and approved the final manuscript.

Funding

This research is funded by the National Institute for Health Research (NIHR) Health Technology Assessment (HTA) programme (funder ref: 16/31/98). The views expressed are those of the authors and not necessarily those of the NIHR or the Department of Health and Social Care.

Availability of data and materials

The datasets analysed during the current study will be available from the corresponding author on reasonable request.

Competing interest

None declared

Patient consent for publication

Not required.

For peer review only - http://bmjopen.bmj.com/site/about/guidelines.xhtml

Licence Statement

I, the Submitting Author has the right to grant and does grant on behalf of all authors of the Work (as defined in the below author licence), an exclusive licence and/or a non-exclusive licence for contributions from authors who are: i) UK Crown employees; ii) where BMJ has agreed a CC-BY licence shall apply, and/or iii) in accordance with the terms applicable for US Federal Government officers or employees acting as part of their official duties; on a worldwide, perpetual, irrevocable, royalty-free basis to BMJ Publishing Group Ltd ("BMJ") its licensees and where the relevant Journal is co-owned by BMJ to the co-owners of the Journal, to publish the Work in BMJ Open and any other BMJ products and to exploit all rights, as set out in our licence.

Ethics approval

The study received North of Scotland Research Ethics Committee (REC) approval on October 29th 2018 and Health Research Authority approval on November 14th 2018. Any amendments to the protocol will be reported accordingly to the regulatory bodies.

Author e-mail and ORCID IDs http://orcid.org/

Penny Seume (PS):	penny.seume@bristol.ac.uk	0000-0001-9407-5828
Scott Bevan (SB):	scott.t.bevan@gmail.com	0000-0002-4567-5111
Grace Young (GY):	grace.young@bristol.ac.uk	0000-0002-5210-1183
Jenny Ingram (JI):	jenny.ingram@bristol.ac.uk	0000-0003-2366-008X
Clare Clement (CClem):	c.clement@bristol.ac.uk	0000-0002-5555-433X
Christie Cabral (CCab):	christie.cabral@bristol.ac.uk	
Patricia Lucas (PL):	patricia.lucas@bristol.ac.uk	0000-0002-0469-8085
Elizabeth Beech (EB):	Elizabeth.beech@nhs.net	
Jodi Taylor (JT):	j.taylor@bristol.ac.uk	0000-0001-7171-8923
Jeremy Horwood (JH):	jj.horwood@bristol.ac.uk	0000-0001-7092-4960
Padraig Dixon (PD):	Padraig.dixon@bristol.ac.uk	0000-0001-5285-409X
Martin Gulliford (MG):	martin.gulliford@kcl.ac.uk	0000-0003-1898-9075
Nick Francis (NF):	nick.francis@soton.ac.uk	
Sam Creavin (SC) :	sam.creavin@bristol.ac.uk	0000-0002-6772-7111
Alastair Hay (AH):	alastair.hay@bristol.ac.uk	0000-0003-3012-375X
Athene Lane (AL):	athene.lane@bristol.ac.uk	0000-0002-7578-4925
Peter Blair (PB):	p.s.blair@bris.ac.uk	0000-0002-7832-8087

For peer review only

For peer review only - http://bmjopen.bmj.com/site/about/guidelines.xhtml

References

1. Smith DRM, Dolk FCK, Pouwels KB, Christie M, Robotham JV, Smieszek T. Defining the appropriateness and inappropriateness of antibiotic prescribing in primary care. J Antimicrob Chemother. 2018 Feb 1;73(suppl_2):ii11-ii18.
2. Bou-Antoun S, Costelloe C, Honeyford K, Mazidi M, Hayhoe BWJ, Holmes A, Johnson AP, Aylin P. Age-related decline in antibiotic prescribing for uncomplicated respiratory tract infections in primary care in England following the introduction of a national financial incentive (the Quality Premium) for health commissioners to reduce use of antibiotics in the community: an interrupted time series analysis. J Antimicrob Chemother. 2018 Oct 1;73(10):2883-2892.
3. Vodicka TA, Thompson M, Lucas P, Heneghan C, Blair PS, Buckley DI, Redmond N and Hay AD. Reducing antibiotic prescribing for children with respiratory tract infections in primary care: a systematic review. Br J Gen Pract 2013 Jul;63(612): e445-54.
4. Davies SC , Gibbens N . UK five year antimicrobial resistance strategy 2013 to 2018. London: Department of Health, Crown Copyright 2013. https://assets.publishing.service.gov.uk/government/uploads/system/uploads/attachment_data/file/244058/20130902_UK_5_year_AMR_strategy.pdf
5. Horwood, J, Cabral, C, Hay, A & Ingram, J, 2015, 'Primary healthcare practitioners' diagnostic and antibiotic prescribing decisions in children's consultations for respiratory tract infections'. British Journal of General Practice. 66 (644), e207-e213
6. Cabral C, Ingram J, Hay AD, Horwood J; TARGET team. "They just say everything's a virus"—Parent's judgment of the credibility of clinician communication in primary care consultations for respiratory tract infections in children: A qualitative study. Patient Educ Couns. 2014 May;95(2):248-53.
7. Hay AD, Redmond NM, Turnbull S, Christensen H, Thornton H, Little P, Thompson M, Delaney B, Lovering AM, Muir P, et al. Development and internal validation of a clinical rule to improve antibiotic use in children presenting to primary care with acute respiratory tract infection and cough: a prognostic cohort study. Lancet Respir Med. 2016;11:902-10.
8. Blair PS, Turnbull S, Ingram J, Redmond N, Lucas PJ, Cabral C, Hollinghurst S, Dixon P, Peters T, Horwood J, Little P, Francis NA, Gilbertson A, Jameson C, Hay AD. Feasibility cluster randomised controlled trial of a within-consultation intervention to reduce antibiotic prescribing for children presenting to primary care with acute respiratory tract infection and cough. BMJ Open. 2017 May 9;7(5):e014506.
9. NIHR: Annual Efficient Studies funding calls for CTU projects. https://www.nihr.ac.uk/documents/ad-hoc-funding-calls-for-ctu-projects/20141

For peer review only - http://bmjopen.bmj.com/site/about/guidelines.xhtml

10. ePACT2 prescribing data held by NHS Prescription Services. https://www.nhsbsa.nhs.uk/epact2
11. Kontopantelis E, Stevens RJ, Helms PJ, Edwards D, Doran T, Ashcroft DM. Spatial Distribution of Clinical Computer Systems in Primary Care in England in 2016 and Implications for Primary Care Electronic Medical Record Databases: A Cross-Sectional Population Study. BMJ Open. 2018 Feb 28;8(2):e020738.
12. Lucas PJ, Ingram J, Redmond NM, Cabral C, Turnbull SL, Hay AD. Development of an Intervention to Reduce Antibiotic Use for Childhood Coughs in UK Primary Care Using Critical Synthesis of Multi-Method Research. BMC Med Res Methodol 2017 Dec 28;17(1):175.
13. Cabral C, Ingram J, Redmond N, Horwood J, Blair P, Hollinghurst S, Hay A, Lucas P. Caring for children with coughs: Information and advice for parents. https://child-cough.bristol.ac.uk, University of Bristol, 2016.
14. Hemming K, Eldridge S, Forbes G et al. 2017. How to design efficient cluster randomised trials. BMJ 2017;358:j3064
15. Gulliford, M.C., van Staa, T.P., McDermott L. et al. 2014. Cluster randomized trials utilizing primary care electronic health records: methodological issues in design, conduct, and analysis (eCRT study).
16. Eldridge SM, Ashby D and Kerry S. Sample size for cluster randomized trials: effect of coefficient of variation of cluster size and analysis method. Int J of Epidemiology 2006;35:1292–1300
17. May CR, Mair F, Finch T, et al. Development of a theory of implementation and integration: Normalization Process Theory. Implementation Science: IS 2009; 4:29-29.
18. Murray E, Treweek S, Pope C, et al. Normalisation process theory: a framework for developing, evaluating and implementing complex interventions. BMC Medicine 2010; 8:63-63.
19. Sandelowski M. Sample size in qualitative research. Research in Nursing & Health 1995; 18(2):179-83.
20. Schulz KF, Altman DG, Moher D; CONSORT Group. CONSORT 2010 Statement: updated guidelines for reporting parallel group randomised trials. BMJ 2010;340:c332
21. QSR International. What is NVivo? | NVivo 2019 [Available from: https://www.qsrinternational.com/nvivo-qualitative-data-analysis-software/about/nvivo.
22. Braun V, Clarke V. Using thematic analysis in psychology. Qualitative Research in Psychology 2006; 3(2):77-101.

For peer review only

23. Boyatzis R. Transforming qualitative information: Thematic analysis and code development. Thousand Oaks, CA: Sage, 1998.
24. Charmay K. Constructing grounded theory: a practical guide through qualitative analysis. London: Sage, 2006.
25. Collett C. Seeking informed consent for simple and efficient trials in the NHS. Draft guidance and responses: Health Research Authority, February 2016.
26. Zwarenstein M, Treweek S, Gagnier J.J et al. Improving the reporting of pragmatic trials: an extension of the CONSORT statement. BMJ 2008, 337:a2390

For peer review only

For peer review only - http://bmjopen.bmj.com/site/about/guidelines.xhtml

Table 1: Text for algorithm result

Algorithm result	Pop-up text
Very low risk group	Very reassuring CHICO score: 0 or 1 CHICO predictors: >99.6% of children will recover from this illness with home care. Consider a no or delayed antibiotic prescribing strategy. CHICO leaflet and letter covers common concerns and safety netting advice.
Average risk group	Reassuring CHICO score: 2 or 3 CHICO predictors: >98% of children will recover from this illness with home care. Consider no or delayed antibiotic prescribing strategy. CHICO leaflet and letter covers common concerns and safety netting advice.
Elevated risk group	Safety netting needed: 4+ CHICO predictors: This is more than average, but >87% of children will still recover from this illness with home care. Highlight SAFETY NETTING advice in CHICO leaflet.

For peer review only

For peer review only - http://bmjopen.bmj.com/site/about/guidelines.xhtml

Table 2: Detailed Study Outcomes

**Primary Outcomes**	
P1)	Whether the CHICO intervention decrease the number of dispensed prescriptions for oral amoxicillin and macrolide antibiotics[1] (efficacy comparison).
P2)	Whether the CHICO intervention result in no increase in hospital admissions[2] for children with a hospital diagnosis of RTI (non-inferiority comparison).
**Secondary Outcomes**	
S1)	Whether the CHICO intervention results in no change in the Emergency Department (ED) attendance rates[3] of children with a diagnosis of RTI.
S2)	The costs to the NHS of using the CHICO intervention (health economic outcome).
S3)	Whether there is any intervention effect modified by the number of locums used in the practice (treatment interaction).
S4)	Whether there is any intervention effect modified by the practices' prior antibiotic prescribing rate (treatment interaction).
S5)	Whether the effects of the CHICO intervention differ between practices with or without nurse prescribers (treatment interaction)[4].
S6[5])	Whether the effects of the CHICO intervention differ between practices with 1 site versus multiple sites (branches) at each practice (treatment interaction).
S7[5])	Whether the effects of the CHICO intervention differ between practices with follow up prior to Covid-19 pandemic and during the Covid-19 pandemic (treatment interaction).
S8[5])	Whether the effects of the CHICO intervention differ in areas of high/low deprivation.
S9)	Whether the effects of the CHICO intervention differ within child age groups.
S10)	Whether the use of the CHICO intervention varies between practices (adherence) and over time (seasonal differences) and the influence this has on the dispensing rates.
S11)	Whether the embedded CHICO intervention is acceptable to, and used by, primary care clinicians (GPs and practice nurses).

1 The dispensing rate, calculated by adding the number of amoxicillin and macrolide antibiotics dispensed over the follow up year divided by the number of children aged 0-9 years (median monthly list size) at each practice over the 12-month follow up period.
2. The rate of hospital admission for RTI amongst children aged 0-9 years using the same denominator as above.
3. This is a secondary outcome already collected from practices by CCGs.
4. If a large majority of practices have nurse prescribers then we may look at this as a continuous percentage of nurse prescribers, out of all GP and nurse prescribers.
5. Added after the trial began, due to unforeseen circumstances including more variability in practices than we first anticipated. Therefore, these do not match those listed in the trial registration.

Figure 1: Trial Schematic

For peer review only - http://bmjopen.bmj.com/site/about/guidelines.xhtml

Figure 1: Trial schematic

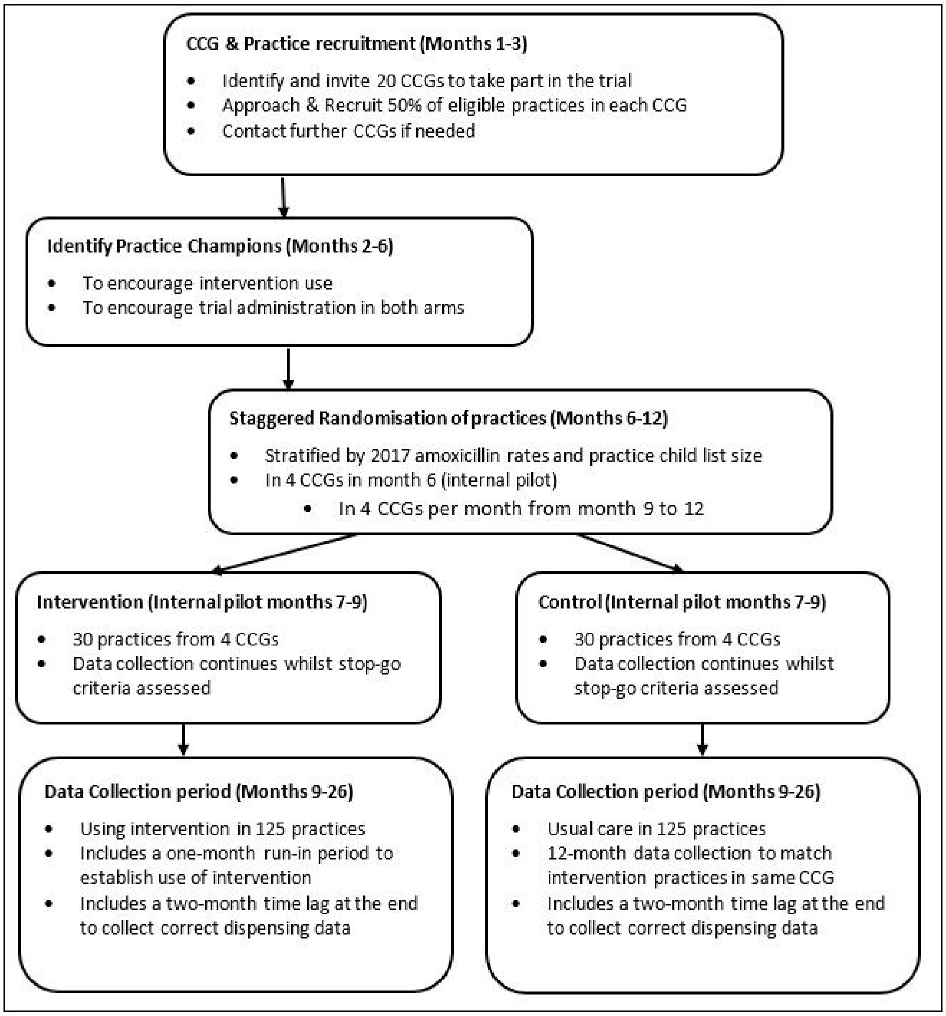

Figure 1: Trial Scematic

58x66mm (300 x 300 DPI)

STANDARD PROTOCOL ITEMS: RECOMMENDATIONS FOR INTERVENTIONAL TRIALS

SPIRIT 2013 Checklist: Recommended items to address in a clinical trial protocol and related documents*

Section/item	Item No	Description	Addressed on page number
**Administrative information**			
Title	1	Descriptive title identifying the study design, population, interventions, and, if applicable, trial acronym	Title Page (p1)
Trial registration	2a	Trial identifier and registry name. If not yet registered, name of intended registry	4
	2b	All items from the World Health Organization Trial Registration Data Set	4
Protocol version	3	Date and version identifier	3
Funding	4	Sources and types of financial, material, and other support	15
Roles and responsibilities	5a	Names, affiliations, and roles of protocol contributors	1
	5b	Name and contact information for the trial sponsor	15
	5c	Role of study sponsor and funders, if any, in study design; collection, management, analysis, and interpretation of data; writing of the report; and the decision to submit the report for publication, including whether they will have ultimate authority over any of these activities	Not applicable
	5d	Composition, roles, and responsibilities of the coordinating centre, steering committee, endpoint adjudication committee, data management team, and other individuals or groups overseeing the trial, if applicable (see Item 21a for data monitoring committee)	8,10,12,13,14

For peer review only

**Introduction**			
Background and rationale	6a	Description of research question and justification for undertaking the trial, including summary of relevant studies (published and unpublished) examining benefits and harms for each intervention	Abstract (p3),5
	6b	Explanation for choice of comparators	7-8
Objectives	7	Specific objectives or hypotheses	6
Trial design	8	Description of trial design including type of trial (eg, parallel group, crossover, factorial, single group), allocation ratio, and framework (eg, superiority, equivalence, noninferiority, exploratory)	6
**Methods: Participants, interventions, and outcomes**			
Study setting	9	Description of study settings (eg, community clinic, academic hospital) and list of countries where data will be collected. Reference to where list of study sites can be obtained	6
Eligibility criteria	10	Inclusion and exclusion criteria for participants. If applicable, eligibility criteria for study centres and individuals who will perform the interventions (eg, surgeons, psychotherapists)	6-7
Interventions	11a	Interventions for each group with sufficient detail to allow replication, including how and when they will be administered	7
	11b	Criteria for discontinuing or modifying allocated interventions for a given trial participant (eg, drug dose change in response to harms, participant request, or improving/worsening disease)	Not Applicable
	11c	Strategies to improve adherence to intervention protocols, and any procedures for monitoring adherence (eg, drug tablet return, laboratory tests)	Not Applicable
	11d	Relevant concomitant care and interventions that are permitted or prohibited during the trial	6
Outcomes	12	Primary, secondary, and other outcomes, including the specific measurement variable (eg, systolic blood pressure), analysis metric (eg, change from baseline, final value, time to event), method of aggregation (eg, median, proportion), and time point for each outcome. Explanation of the clinical relevance of chosen efficacy and harm outcomes is strongly recommended	3,9,12,Box 3 (p22)
Participant timeline	13	Time schedule of enrolment, interventions (including any run-ins and washouts), assessments, and visits for participants. A schematic diagram is highly recommended (see Figure)	Box 2 (p21)

For peer review only - http://bmjopen.bmj.com/site/about/guidelines.xhtml

Sample size	14	Estimated number of participants needed to achieve study objectives and how it was determined, including clinical and statistical assumptions supporting any sample size calculations	10-11
Recruitment	15	Strategies for achieving adequate participant enrolment to reach target sample size	6,10
**Methods: Assignment of interventions (for controlled trials)**			
Allocation:			
Sequence generation	16a	Method of generating the allocation sequence (eg, computer-generated random numbers), and list of any factors for stratification. To reduce predictability of a random sequence, details of any planned restriction (eg, blocking) should be provided in a separate document that is unavailable to those who enrol participants or assign interventions	3,8-9
Allocation concealment mechanism	16b	Mechanism of implementing the allocation sequence (eg, central telephone; sequentially numbered, opaque, sealed envelopes), describing any steps to conceal the sequence until interventions are assigned	8,12
Implementation	16c	Who will generate the allocation sequence, who will enrol participants, and who will assign participants to interventions	8-9
Blinding (masking)	17a	Who will be blinded after assignment to interventions (eg, trial participants, care providers, outcome assessors, data analysts), and how	9
	17b	If blinded, circumstances under which unblinding is permissible, and procedure for revealing a participant's allocated intervention during the trial	Not applicable
**Methods: Data collection, management, and analysis**			
Data collection methods	18a	Plans for assessment and collection of outcome, baseline, and other trial data, including any related processes to promote data quality (eg, duplicate measurements, training of assessors) and a description of study instruments (eg, questionnaires, laboratory tests) along with their reliability and validity, if known. Reference to where data collection forms can be found, if not in the protocol	7-9
	18b	Plans to promote participant retention and complete follow-up, including list of any outcome data to be collected for participants who discontinue or deviate from intervention protocols	

For peer review only - http://bmjopen.bmj.com/site/about/guidelines.xhtml

Data management	19	Plans for data entry, coding, security, and storage, including any related processes to promote data quality (eg, double data entry; range checks for data values). Reference to where details of data management procedures can be found, if not in the protocol	13
Statistical methods	20a	Statistical methods for analysing primary and secondary outcomes. Reference to where other details of the statistical analysis plan can be found, if not in the protocol	12
	20b	Methods for any additional analyses (eg, subgroup and adjusted analyses)	12
	20c	Definition of analysis population relating to protocol non-adherence (eg, as randomised analysis), and any statistical methods to handle missing data (eg, multiple imputation)	12
**Methods: Monitoring**			
Data monitoring	21a	Composition of data monitoring committee (DMC); summary of its role and reporting structure; statement of whether it is independent from the sponsor and competing interests; and reference to where further details about its charter can be found, if not in the protocol. Alternatively, an explanation of why a DMC is not needed	10,13-14
	21b	Description of any interim analyses and stopping guidelines, including who will have access to these interim results and make the final decision to terminate the trial	Not applicable
Harms	22	Plans for collecting, assessing, reporting, and managing solicited and spontaneously reported adverse events and other unintended effects of trial interventions or trial conduct	9-10
Auditing	23	Frequency and procedures for auditing trial conduct, if any, and whether the process will be independent from investigators and the sponsor	10
**Ethics and dissemination**			
Research ethics approval	24	Plans for seeking research ethics committee/institutional review board (REC/IRB) approval	3
Protocol amendments	25	Plans for communicating important protocol modifications (eg, changes to eligibility criteria, outcomes, analyses) to relevant parties (eg, investigators, REC/IRBs, trial participants, trial registries, journals, regulators)	15

Consent or assent	26a	Who will obtain informed consent or assent from potential trial participants or authorised surrogates, and how (see Item 32)	Not applicable
	26b	Additional consent provisions for collection and use of participant data and biological specimens in ancillary studies, if applicable	Not applicable
Confidentiality	27	How personal information about potential and enrolled participants will be collected, shared, and maintained in order to protect confidentiality before, during, and after the trial	8
Declaration of interests	28	Financial and other competing interests for principal investigators for the overall trial and each study site	Not applicable
Access to data	29	Statement of who will have access to the final trial dataset, and disclosure of contractual agreements that limit such access for investigators	15
Ancillary and post-trial care	30	Provisions, if any, for ancillary and post-trial care, and for compensation to those who suffer harm from trial participation	Not applicable
Dissemination policy	31a	Plans for investigators and sponsor to communicate trial results to participants, healthcare professionals, the public, and other relevant groups (eg, via publication, reporting in results databases, or other data sharing arrangements), including any publication restrictions	14
	31b	Authorship eligibility guidelines and any intended use of professional writers	Not applicable
	31c	Plans, if any, for granting public access to the full protocol, participant-level dataset, and statistical code	Not applicable
**Appendices**			
Informed consent materials	32	Model consent form and other related documentation given to participants and authorised surrogates	Not applicable
Biological specimens	33	Plans for collection, laboratory evaluation, and storage of biological specimens for genetic or molecular analysis in the current trial and for future use in ancillary studies, if applicable	Not applicable

*It is strongly recommended that this checklist be read in conjunction with the SPIRIT 2013 Explanation & Elaboration for important clarification on the items. Amendments to the protocol should be tracked and dated. The SPIRIT checklist is copyrighted by the SPIRIT Group under the Creative Commons "Attribution-NonCommercial-NoDerivs 3.0 Unported" license.

For peer review only - http://bmjopen.bmj.com/site/about/guidelines.xhtml
